# Protection of Human Pancreatic Islets from Lipotoxicity by Modulation of the Translocon

**DOI:** 10.1371/journal.pone.0148686

**Published:** 2016-02-10

**Authors:** R. Cassel, S. Ducreux, M. R. Alam, F. Dingreville, C. Berlé, K. Burda-Jacob, M. A. Chauvin, K. Chikh, L. Païta, R. Al-Mawla, C. Crola Da Silva, J. Rieusset, C. Thivolet, F. Van Coppenolle, A. M. Madec

**Affiliations:** 1 Inserm UMR-U1060 CarMeN Laboratory, University Lyon 1, INRA U1235, INSA-Lyon, Facultés de médecine Charles Mérieux Lyon-Sud, F-69003 Lyon, France; 2 Inserm UMR-U1060 CarMeN Laboratory, University Lyon 1, INRA U1235, INSA-Lyon, Facultés de médecine Rockefeller, F-69003 Lyon, France; 3 Hospices Civils de Lyon, Hôpital Lyon-Sud, Service d’Endocrinologie, Diabétologie et Nutrition, F-69310 Pierre Bénite, France; Communaute d\'Universites et d\'Etablissements Lille Nord de France, FRANCE

## Abstract

Type 2 diabetes is characterized by peripheral insulin resistance and pancreatic beta cell dysfunction. Elevated free fatty acids (FFAs) may impair beta cell function and mass (lipotoxicity). Altered calcium homeostasis may be involved in defective insulin release. The endoplasmic reticulum (ER) is the major intracellular calcium store. Lipotoxicity induces ER stress and in parallel an ER calcium depletion through unknown ER calcium leak channels. The main purposes of this study is first to identify one of these channels and secondly, to check the opportunity to restore beta cells function (i.e., insulin secretion) after pharmacological inhibition of ER calcium store depletion. We investigated the functionality of translocon, an ER calcium leak channel and its involvement on FFAs-induced alterations in MIN6B1 cells and in human pancreatic islets. We evidenced that translocon acts as a functional ER calcium leak channel in human beta cells using anisomycin and puromycin (antibiotics), respectively blocker and opener of this channel. Puromycin induced a significant ER calcium release, inhibited by anisomycin pretreatment. Palmitate treatment was used as FFA model to induce a mild lipotoxic effect: ER calcium content was reduced, ER stress but not apoptosis were induced and glucose induced insulin secretion was decreased in our beta cells. Interestingly, translocon inhibition by chronic anisomycin treatment prevented dysfunctions induced by palmitate, avoiding reticular calcium depletion, ER stress and restoring insulin secretion. Our results provide for the first time compelling evidence that translocon actively participates to the palmitate-induced ER calcium leak and insulin secretion decrease in beta cells. Its inhibition reduces these lipotoxic effects. Taken together, our data indicate that TLC may be a new potential target for the treatment of type 2 diabetes.

## Introduction

Type 2 diabetes is a worldwide multifactorial disease characterized by peripheral insulin resistance and pancreatic beta cell dysfunction [1,2]. This heterogeneous disease results from a complex environment-inheritance interaction [3]. Free fatty acid (FFA) levels are an independent predictor of future type 2 diabetes [4]. High intake of saturated fatty acids (FFAs) has also been linked to higher risks of type 2 diabetes [5]. Previous studies have shown that palmitate, the most abundant saturated FFA in blood, inhibits insulin signaling in liver, muscle, and fat cells *in vitro* [6–8]. These studies have also shown the deleterious effects of FFAs, collectively termed ‘‘lipotoxicity”, on beta cells *in vitro* [9]. Mainly based on the findings of *in vitro* studies, it was proposed that beta cell lipotoxicity is directly induced by palmitate at least in part via pathways involving endoplasmic reticulum (ER) stress and reactive oxygen species (ROS) [10,11], inflammation [12] and autophagy [13]. Similarly, prolonged *in vivo* infusion of FFAs impairs beta cell function in rodent models and in humans [14]. However, molecular mechanisms by which FFAs induce beta cell dysfunction *in vivo* remain poorly understood.

Chronic exposure to saturated FFAs was shown to enhance the unfolded protein response (UPR) of ER. This phenomenon foremost protects the cells by promoting the folding of proteins in the ER lumen and/or their degradation by the 26S proteasome. On the contrary, a prolonged UPR could also trigger apoptosis if ER function is not preserved or restored [15–17]. Due to high insulin protein production, beta cells are particularly susceptible to the activation of UPR and ER stress. Particularly, the phosphorylation of PERK and the induction of the transcription factor CHOP is a key feature for the saturated FFA-induced progression to apoptosis [18,19]. Relevance of these *in vitro* models to human disease was confirmed by the enhanced expression of ER stress markers in beta cells of type 2 diabetic patients [16,17] and by the recent clinical trial of an ER stress reducing drug (phenylbutyric acid), which improved beta cell functions caused by prolonged hyperlipidemia [20]. Thus, how mechanically ER stress is induced by saturated FFAs is still an unanswered question. ER stress is due to the accumulation of unfolded proteins within the ER and/or the depletion of calcium stores [21,22], both leading to apoptosis.

Altered cellular calcium homeostasis may be involved in defective insulin release [23]. Calcium plays an important role in ER stress and in UPR, and the regulation of ER calcium homeostasis is closely linked to precise control of ER calcium leak channels permeability. Indeed, ER calcium concentration is an equilibrium between ER calcium entry through SERCA pumps and calcium leak via opened calcium leak channels. These channels, involved in ER stress transduction pathways, are not yet characterized in pancreatic beta cells. Gwiazda *et al*. pointed out a direct link between ER calcium decrease with ER stress in human islets and MIN6 murine beta cells [23]. However, the molecular mechanisms of calcium dysregulation during ER stress in beta cells are still unknown. Therefore, the modulation of ER calcium permeability during UPR could be a useful way to better understand ER calcium involvement in UPR in physiological and pathological conditions especially in type 2 diabetes.

In previous studies [24,25], we have shown that translocon, a complex involved in protein translocation during translation [26], is an ER calcium leak channel in human cancerous prostatic cells. Translocon has also been demonstrated to act as a calcium leak channel by other groups and in several cell types: in Xenopus oocytes [27]; in rat liver microsomal vesicles [28]; in Sec61 proteoliposomes obtained from dog pancreas [29,30] and in mouse pancreatic acinar cells [31]. Recently, we further demonstrated that ER calcium depletion by thapsigargin (an inhibitor of SERCA pumps and an ER stress inducer) occurs mainly via translocon, which is also implied in the unfolded proteins retro-translocation for their degradation by the 26S proteasome [32]. This process is called ERAD for ER-associated degradation [33]. In parallel to ER protein retro-translocation through translocon, calcium may follow the same way. In this study, we investigated for the first time the functionality of translocon in the calcium homeostasis of pancreatic beta cells and its involvement in palmitate-induced alterations of both human pancreatic islets and murine beta cells.

## Material and Methods

### Islet culture

Human pancreatic islets from 8 non-diabetic donors (Table 1) were obtained through the Geneva European Consortium for islet transplantation (ECIT) and Grenoble Cell Therapy Unit (UMTC). The use of human islet preparations for experimental research was approved by the Institutional Review Board for clinical research of the Departments of Neurology, Dermatology, Anesthesiology and Surgery of the University Hospital of Geneva (CER Nr. 05–028). The Geneva University Hospital ethical institution waived the need for consent from the donor. Islets were used for experimental research only when not suitable for clinical purposes and with the intention to be destroyed. In such cases, obtaining informed consent is not mandatory in Europe. Tissues samples were not procured from a tissue bank. Our ethical review board of Lyon-Sud Medicine Faculty specifically approved this study.

**Table 1:** Characteristics of human islet donors.

N°	Gender	Age (y)	BMI (kg/m^2^)
1	M	48	23.2
2	M	58	29.3
3	F	54	24.7
4	M	54	26.7
5	M	53	27.8
6	M	39	22.0
7	F	38	23.0
8	M	39	22.9
**Moyenne**		**47.9**	**25.0**
**SEM**		2.9	0.9

Islets (purity 80%) were processed as described previously [34], and cultured in a DMEM medium containing 5.5 mM glucose and 5% (vol./vol.) SVF. For treatments, islets were incubated with either BSA or palmitate/BSA (6:1, 500μM) during 48h, in presence or absence of anisomycin (200nM or 500nM).

### MIN6B1 culture

Murine beta cells MIN6B1 (provided by Dr. Jun-Ichi Miyazaki, OUMS, Osaka, Japan) were cultured in DMEM medium containing 15% SVF, 2 mM glutamine, 100U/ml penicillin, and 100mg/l streptomycin.

### Insulin secretion study

At the end of the 48h incubation period, islets were kept at 37°C for 45 min in DMEM 5.5 mM glucose and 5% SVF. To assess insulin basal secretion, 50 islets were incubated in KRBH containing 5.5 mM glucose, 5% SVF during 45 min then challenged with 16.7 mM glucose, 5% SVF to assess glucose-stimulated insulin release (GSIS) as previously described [34]. Insulin was quantified using a highly specific immuno-radiometric assay (IRMA) (Bi-insulin IRMA, Cis-Bio International, Gif sur-Yvette France). Islet proteins were used to normalize the secretion values. So insulin expression was expressed in mU/L/μg Prot /H. The quality of the islets was assessed by the high 15.5/5.5 mM Glucose stimulated insulin secretion (4.09 ±0.88 mU/L/μg Prot/H). Due to the high inter-islet variability of insulin secretion (5.5mM glucose basal range: 170–1761 mU/L; 16.5 mM glucose stimulated range: 1410–8786 mU/L), final results were expressed in % of control condition.

### Adenoviral constructions and cell infection

Recombinant adenoviral constructs carrying the cDNA of GRP78 was a generous gift of Tatsuro Koike, (Hokkaido University, Sapporo, Japan). Adenovirus was amplified in HEK293 cells and purified on a cesium chloride gradient (7.5x10^8^ IFU/μL). Recombinant adenoviral constructs carrying the cDNA of Renilla Luciferase (control;10^8^ IFU/mL) and D4-ER (5.7x10^7^ IFU/mL) were generous gifts of Etienne Lefai (INSERM 1060). Infections of MIN6B1 cells were performed in complete medium.

### Transfection

GRP78 silencing was performed using siGRP78 (5′-GGAGCGCAUUGAUACUAGA-3′) in MIN6B1 cells. Control siRNA (siLuc) experiments were performed by transfecting siRNA against luciferase (5′-CUUACGCUGAGUACUUCGA-3′; Dharmacon). Transfection was performed with jetSI ENDO (Polyplus Transfection, Illkirch, France). Protein knockdown was measured after 48H using Western blot assay.

### Intracellular calcium measurements

To measure intracellular calcium concentration dynamics, experiments were performed using the membrane-permeable calcium-sensitive dye Fura-2 AM, as detailed previously [34] in human islets of Langerhans and MIN6B1 cells. All experiments were done in calcium-free medium.

To assess translocon involvement in cellular calcium homeostasis in human pancreatic islets and MIN6B1 cells, thapsigargin (1 μM) was used as SERCAs blocker, leading to a almost full calcium leakage from the ER to the cytosol. The subsequent increased Fura-2 fluorescence was attributed to the ER calcium content. Puromycin (200 μM), a specific translocon opener [24,25], was used alone to determine if translocon opening facilitated ER calcium leak. To verify that this ER calcium liberation was due to translocon calcium activity, we pretreated cells with anisomycin (200 μM), a specific translocon blocker, which abrogated puromycin action. Anisomycin was added 30 minutes before puromycin incubation.

Calcium response to chronic treatment of anisomycin (200 nM) and/or puromycin (200 nM), (IC50 = 10μM; delay = 130s+/-10 for both) was obtained by culturing both MIN6B1 and human islets, in their respective cultured medium, during 48h. The concentrations used, in both acute and chronic conditions, are in accordance with those of our previous work [32]. Anisomycin was added 30 min before puromycin stimulation.

### Measurement of ER luminal Ca^2+^

ER Ca^2+^ in MIN6B1 cells was measured with FRET-based genetic sensors (D1ER or Ad-D4ER) by following live single-cells approach using a widefield microscopy system (Leica Microsystems, France). For Ad-D4ER, cells were infected with the virus particles 48 hours before the experiment while in case of D1ER cells were co-transfected with D1ER plasmid and the respective siRNA in order to use the D1ER as a Ca^2+^ sensor on one hand and as a transfection marker on the other hand. All experiments were performed in a nominal Ca^2+^-free KRB solution with or without thapsigargin addition as described for FURA-2 AM experiments. Both the sensors were excited at a wavelength of 430 nm and emissions were collected at 480 nm and 530 nm. Data was acquired using the MetaFluor Imaging software (Leica Microsystems, France) and analyzed with MS Excel and Graphpad Prism softwares to correct for background and photobleaching.

### Quantification of mRNAs

Total mRNA samples were prepared as previously described [34]. To monitor ER stress markers, we measured mRNA levels of glucose response protein 78 (GRP78), X-box binding protein-1 (spliced XBP1 and total XBP), activating transcription factor 4 (ATF4), and C/EBP homology protein (CHOP). We also measured SEC61α, one of the most expressed subunits of the translocon, to evaluate translocon expression modifications in our conditions. Values were normalized using TATA box binding protein mRNA (TBP). Primers are listed in S1 Table.

### Caspase activation

Caspase -2 to 10- activation was measured with the Homogeneous Caspases Assay (Roche Diagnostics, Manheim, France), following the manufacturer instructions. Human islets were cultured in black-walled-96-well microplates. At the end of treatment, free Rhodamine-110 concentrations were determined in a plate-reading luminometer (λex = 485nm, λem = 510nm).

### Statistical analysis

Data are given as means ± SEM. Statistical analysis were performed using the unpaired Student’s *t*-test (for 2 groups) or by 2-way analysis o f variance (ANOVA) followed by a Newman-Keuls post test (for groups of 3 or more). A value of *p*<0.05 was considered to be statistically significant. Origin computer program (Microcal Software, Inc., Northampton, MA, USA) and Graphpad Prism were used for calcium concentration curve generation and statistical analysis.

## Results

We both used anisomycin and puromycin to respectively close and open translocon as previously described [24,25,32]. As anisomycin is an antibiotic that inhibits protein translation at high concentration, we checked by dose-dependent assay that it does not inhibit protein synthesis during chronic treatments. Anisomycin did not affect protein translation until 0.5 μM and reduced it by more than 50% at 1 and 2 μM (S1 Fig).

### Translocon is functionally involved in beta cell calcium homeostasis

**Fig 1:**
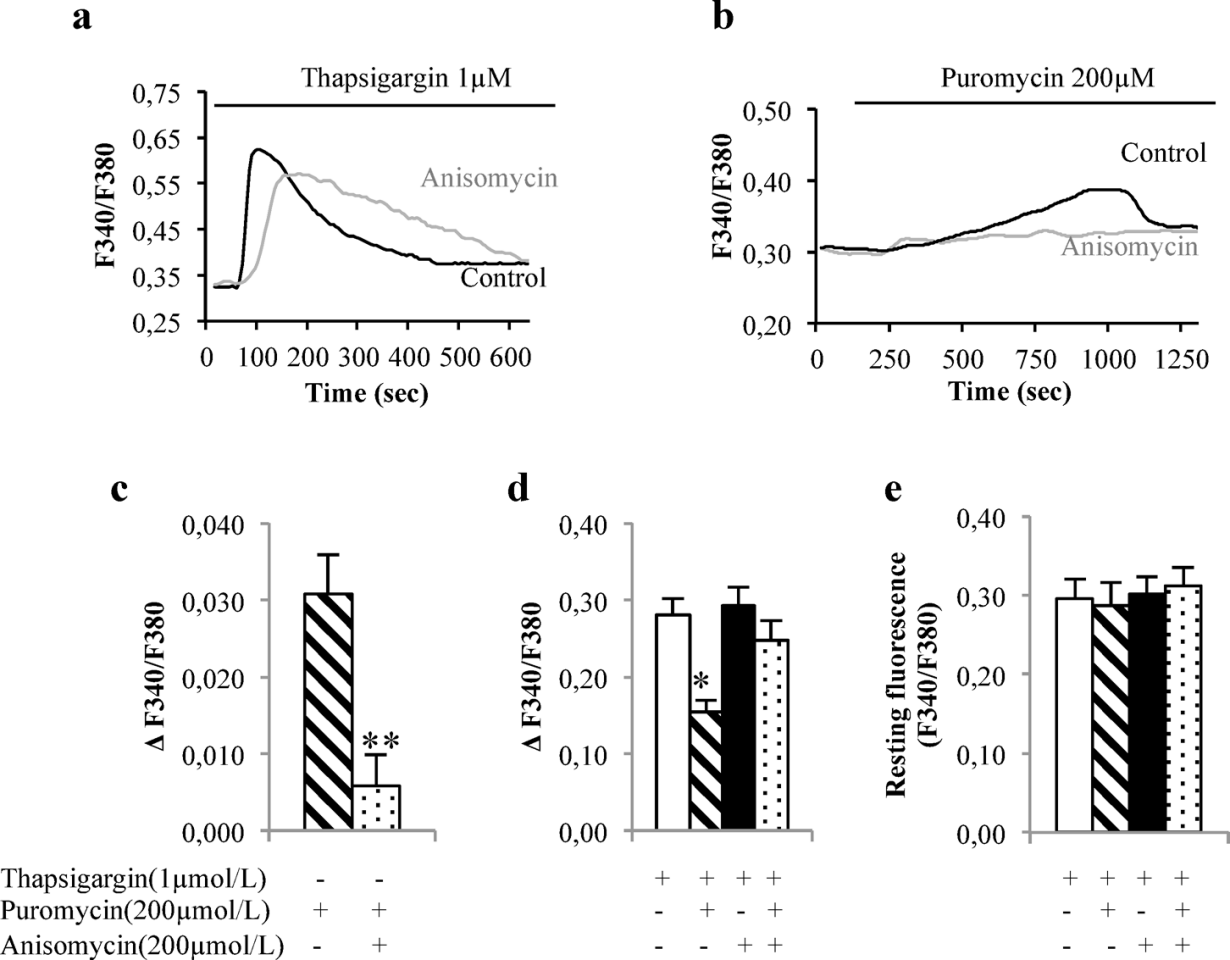
Puromycin and anisomycin acute effects on calcium homeostasis in human islets. Puromycin and anisomcyin effects were evaluated in 4 different human islets preparation (44 different cells) (**a**-**e**). Typical cytosolic calcium traces in response to 200 μM thapsigargin (**a**) or to 1 μM puromycin (**b**) under control conditions and after 30 min incubation with 200 μM anisomycin. Cumulative data of peak cytosolic calcium increases evoked by puromycin responses under control conditions and with anisomycin 200 μM (**c**). Cumulative data of peak cytosolic calcium increases evoked by thapsigargin responses (**d**) and (**e**) resting fluorescence (F340/F380) under control conditions ± puromycin or anisomycin pretreatment. *p<0.05, **p<0.01. Measures were assessed in a calcium-free medium. Preparations were done in duplicate.

ER is a major internal calcium storage organelle in beta cells, which acts as a calcium buffer that regulates calcium-dependent signaling in the cytosol. As we hypothesized that translocon could be involved in global calcium homeostasis of beta cell as an ER calcium leak channel, we first measured puromycin action on ER calcium content in human islets and in MIN6B1 cells. Cytosolic calcium was thus evaluated using the ratiometric Fura-2 AM probe, each ER calcium release would be seen by an increase of the Fura-2 ratio signal. This method is commonly used to appreciate ER calcium content [32]. Another benefit of Fura-2AM is also to directly measure a ratio which allows to quantitatively compare the effects of treatments on the resting cytosolic calcium concentration and on the ER calcium content [32]. In human islets, puromycin induced an increase in cytosolic calcium concentration. This effect was partly abolished by a 30 min pretreatment with anisomycin (Fig 1A and 1C).

We next investigated the reticular calcium pool by comparing the amplitude of calcium peak obtained after adding thapsigargin (Tg) (a SERCA inhibitor) in a calcium-free medium, with puromycin (200μM) and/or 30 minutes pretreatment with anisomycin (200μM) of human islets and MIN6B1. As expected, Tg elicited a transient cytosolic calcium increase in human islets (Fig 1A and 1D). Puromycin pretreatment significantly reduced Tg-induced ER calcium release as compared to control conditions (Fig 1B and 1D). Anisomycin alone was ineffective in Tg response (Fig 1A and 1D). Puromycin, combined with anisomycin, failed to decrease Tg response (Fig 1B and 1D). These results suggest that puromycin pretreatment partly depletes ER calcium content via opening of the translocon leading to a decrease in Tg-induced ER calcium release. We finally checked the impact of puromycin and anisomycin on the resting calcium concentration and found that the basal calcium level remained similar to control conditions in human islets, whatever the pretreatments were (Fig 1E).

Next, we performed similar experiments in MIN6B1 cells, and found that puromycin (200 μM) also induced an ER calcium release, which was inhibited by anisomycin pretreatment (200 μM; S2 Fig). Resting calcium concentrations were not affected by the different pretreatments (S2 Fig). As observed in human islets, the Tg-induced ER calcium release was significantly decreased by puromycin pretreatment as compared to control conditions (S2 Fig).

To exclude the hypothesis that the [Ca^2+^]_ER_ decrease induced by puromycin was mediated by agonist-activated Ca^2+^ release channels, we perfused the IP3 receptor inhibitor (xestospongin C; 3 μM) and the ryanodine receptor inhibitor (ryanodine; 50 μM) in MIN6B1 cells. The S3 Fig illustrates the effect of puromycin alone (100±20.03%; control condition; n = 12 cells) or in the presence of anisomycin (24.78±7.12%; p<0.001 vs. puromycin; n = 14 cells) in MIN6B1 cells. We have not observed a significant modification of puromycin effects on ER calcium release with ryanodine (66.1±9.93% vs. control; p = NS) and xestospongin C (73.03±10.2% vs. control; p = NS) (S3 Fig).

To further characterize the involvement of TLC in ER Ca^2+^ leak, we directly measured the luminal ER Ca^2+^ using genetically-encoded FRET based sensor which are targeted to ER with high efficiency (S4 Fig). After 48 hours of Ad-D4ER infection MIN6B1 cells were pre-treated with anisomycin for 20 minutes and compered with non-treated cells. Anisomycin was kept throughout the acquisition period. After an initial measurement of basal FRET ratio cell were treated with TG in order to estimate the leak-mediated ER Ca^2+^ depletion (S4(A) Fig). In agreement with cytosolic Ca^2+^ data, anisomycin treatment effectively suppressed the TG-induced ER Ca^2+^ depletion as displayed by a significant reduction in the Δ[Ca^2+^]_ER_ before and after TG stimulation (S4(B) Fig). However, these results can be an outcome of a low ER Ca^2+^ content due to anisomycin treatment. In order to answer this question we measured the resting levels of luminal ER Ca^2+^ by using the same data set. Interestingly our analysis revealed an increase in the basal ER Ca^2+^ in anisomycin group (S4(C) Fig) highlighting an effect of anisomycin on the resting ER Ca^2+^ levels probability due to an inhibition of Ca^2+^ leak under basal conditions in this cell type.

Altogether, these results clearly illustrate that translocon is a functional ER calcium leak channel in both human islets and murine beta cells.

### Translocon inhibition partially prevents palmitate-induced reticular calcium depletion

**Fig 2:**
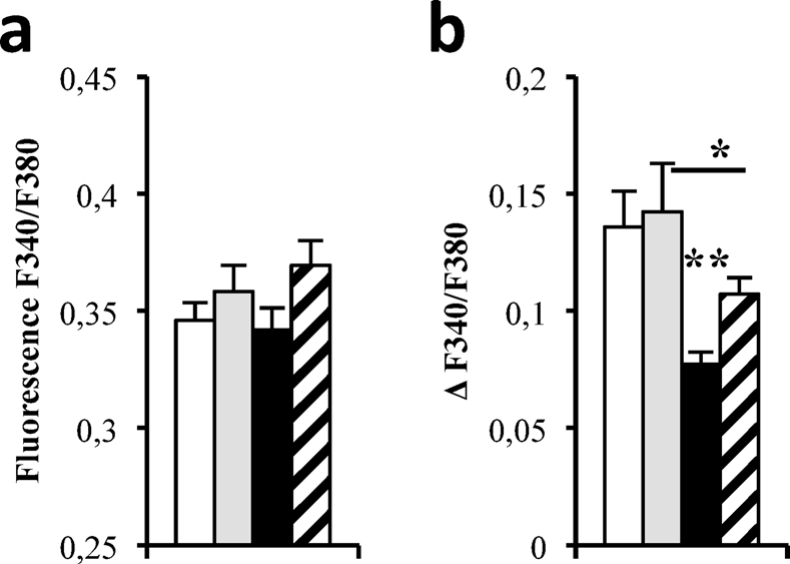
Translocon inhibition partially prevents palmitate-induced reticular calcium depletion. Quantification of fluorescence ratio (F340/F380) of resting calcium (**a**) and of reticular calcium release induced by 1 μM thapsigargin (**b**) in 4 different human islets preparations (11 cells per preparation) under 48h BSA (white bar) + anisomycin (grey bars and palmitate (black bar) conditions + anisomycin (hatched bars). *p<0.05, **p<0.01.

As mentioned earlier, ER stress is linked to ER calcium depletion. In this context, we wanted to know if pharmaceutical modulation of translocon opening could modify ER calcium content in a long-term manner in both control and palmitate-treated conditions. To establish the characteristics of the intracellular calcium pools, we first analyzed the resting concentration in cytosol of human islets exposed to palmitate (500 μM) with or without anisomycin (200 nM). We observed that chronic treatment (48h) with palmitate did not change resting calcium as compared to control conditions in human islets (Fig 2A). Chronic addition of anisomycin has not altered resting calcium in control condition in human islets (Fig 2A). Resting calcium in human islets exposed to palmitate and anisomycin was similar compared to palmitate condition (Fig 2A and 2C). After Tg stimulation, reticular calcium concentration was reduced in human islets exposed to chronic palmitate treatment (Fig 2B). Addition of anisomycin partially restored reticular calcium content (Fig 2B). Anisomycin did not modify ER calcium content in basal conditions (Fig 2B and 2D).

Similar results were obtained in murine pancreatic beta cells MIN6B1 treated by palmitate (S5 Fig).

We previously modulated the permeability of translocon using pharmacological compounds. One way would have been to inhibit Sec61α expression using siRNA. Nevertheless, as Sec61α is one of the major subunit of the translocon, the decrease of its expression is deleterious for protein synthesis and cell survival. So, as shown in S6 Fig, we then overexpressed GRP78 (also called as Bip) using adenoviruses in Min6B1 cells. GRP78 is present in the ER lumen where it interacts with many other proteins in the lumen and/or ER membrane including translocon [11,17]. By binding the luminal side of translocon it has been proposed to block the ER Ca^2+^ leak [32,33]. We used a Renilla Luciferase-adenovirus as a control. Resting calcium concentration is not modified by GRP78 overexpression neither in BSA nor in palmitate condition (S6 Fig). ER calcium content was appreciated using TG response. As expected, palmitate treatment decreases ER calcium concentration (S6 Fig). GRP78 overexpression is here ineffective to significantly restore ER calcium content. Quantitative analysis of GRP78 expression was appreciated using Western blot (S7 Fig).We also checked siRNA-mediated knock-down of GRP78 (S8 Fig). We did not notice any differences between ER calcium content in control and in siRNA treated cells using ad-D4ER or Fura-2 (data not shown).

### Translocon expression is not modified by palmitate

The translocon complex is composed of several protein complexes. One of which is Sec61α and is involved in the structure of the pore. Here, we measured whether palmitate (500 μM) and/or anisomycin (200 nM) treatments could alter the SEC61α mRNA expression. Palmitate did not modify SEC61α mRNA expression in human islets (91.38±3.89% vs. control; p = NS). Anisomycin alone or in combination with palmitate did not modify Sec61α mRNA expression (94.18±1.97% vs. control; p = NS). Anisomycin in basal conditions did not have any effect on this expression (109.37±22.27% vs. control). These data suggest that the anisomycin capacity to restore ER calcium content under palmitate stress could not be attributed to major variations in the translocon mRNA expression.

### Translocon inhibition partially modulates palmitate-induced ER stress

As palmitate is a cause of ER stress in beta cells, we investigated the capacity of anisomycin (200 nM) to reduce the ER stress in pancreatic beta cells in response to palmitate (500 μM).

**Fig 3:**
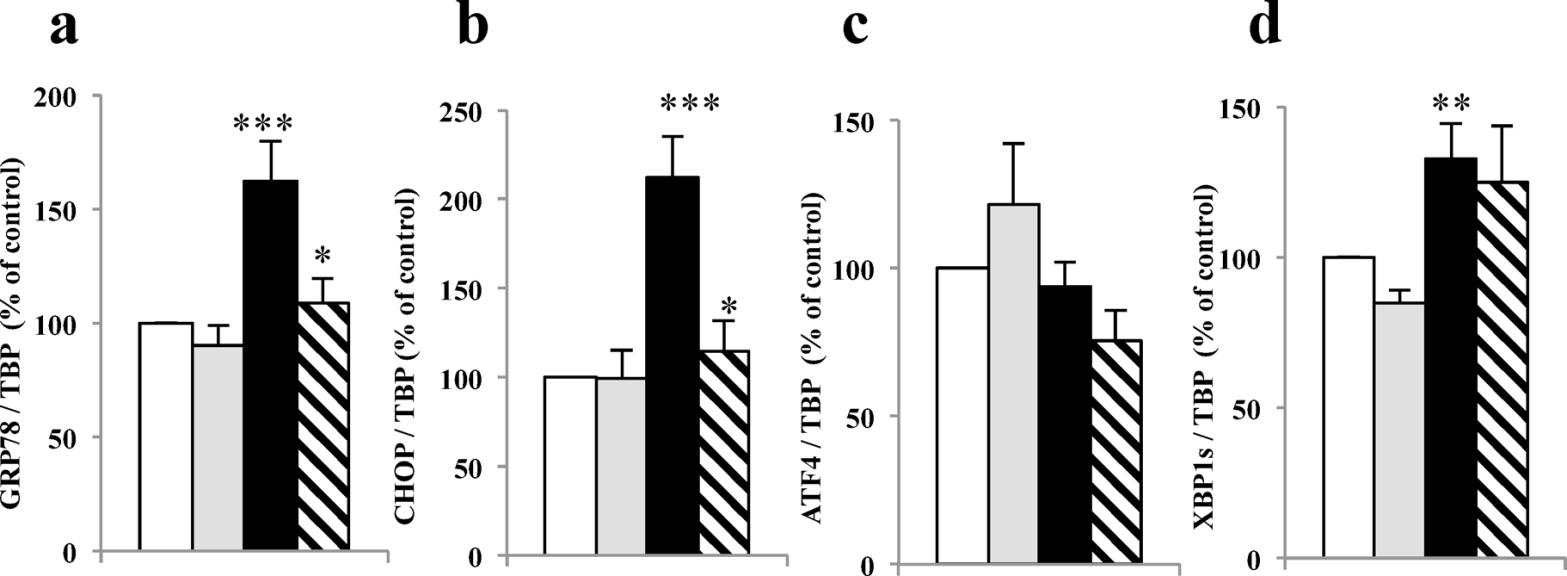
Translocon inhibition partially modulates palmitate-induced ER stress. ER stress mRNA expression markers were measured after 48h BSA (white bar) + anisomycin (grey bars) or palmitate (black bar) + anisomycin (hatched bars) treatment in 5 different human islet preparations. ***p<0.001;**p<0.01;*p<0.05.

Human islets, cultured for 48h in presence of palmitate, showed a significant increase of both GRP78 (Fig 3A), CHOP (Fig 3B) and XBP1s (Fig 3D) mRNA levels, whereas ATF4 was not modified (Fig 3C). Anisomycin had no effect on ER stress markers mRNA expression in basal conditions but significantly reduces GRP78 and CHOP mRNA levels in palmitate conditions in human islets (Fig 3A and 3B).

### Palmitate treatment does not activate caspase activity in human islets

To assess survival of cells, we next evaluated caspase activity via free Rhodamine-110 measurement in human islets cultured in BSA or palmitate (500 μM) conditions in presence or absence of anisomycin (200 nM). No caspase activation was significantly induced by palmitate in the tested set of human islets in our culture conditions (119.97±16.96% vs. control; p = NS) and anisomycin treatment did not modify caspase level in both basal and palmitate conditions (103.23±8.6% and 143.34±16.60% vs. control respectively; p = NS).

### Anisomycin restores GSIS in palmitate condition in human islets

**Fig 4:**
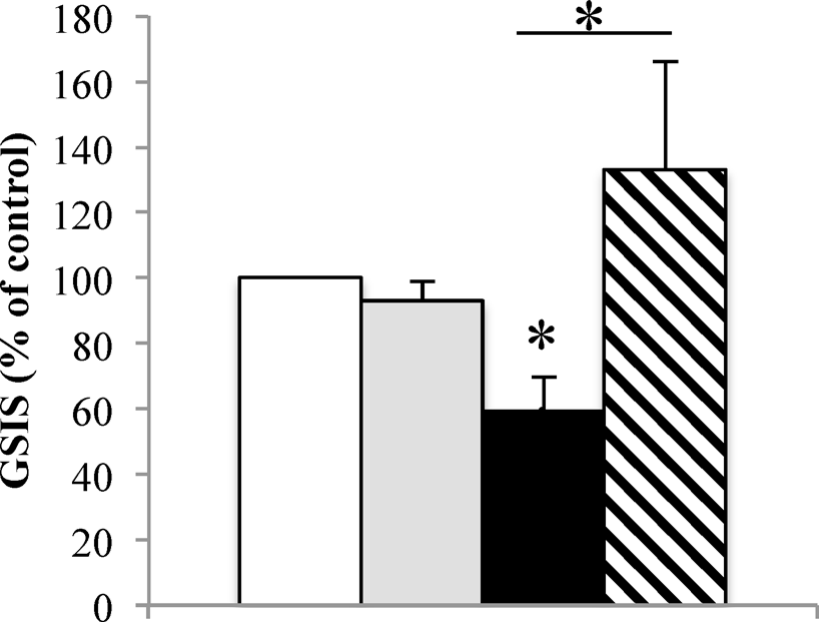
Anisomycin restores GSIS in palmitate condition in human islets. GSIS were measured after 48h BSA (white bar) + anisomycin (200 nM; grey bars) or palmitate (black bar) treatment + anisomycin (200 nM; hatched bars) in 6 human islet different preparations. *p<0.05.

We finally measured the beta cell function in our conditions. Palmitate (500 μM) significantly reduced the glucose-stimulated insulin secretion (GSIS) in human islets (59.33±10.41% vs. control; p<0.01) (Fig 4). Interestingly, the inhibition of translocon opening by anisomycin treatment completely prevented the palmitate-induced dysfunction of human islets (133.17±32.90% vs. 59.33±10.41% p<0.05) (Fig 4). Anisomycin did not modified GSIS in basal conditions (92.96±6.01% vs. control; p = NS).

## Discussion

In type 2 diabetes, prolonged exposure to FFAs contributes to insulin resistance and beta cell dysfunction [35, 36]. These data are reinforced by the last results from the large longitudinal prospective study of the EPIC-InterAct case-cohort showing a positive association between plasmatic palmitic acid and incidence of type 2 diabetes [37]. Saturated FFAs, either through oversupply or inappropriate metabolism in the beta cells appear to be one of the primary triggers. Nevertheless, the molecular mechanisms involved in this pathology remain poorly understood [38]. Lipotoxicity is associated with ER calcium depletion and ER stress [39]. An increase in ER calcium efflux first depletes the stores, secondly induces ER stress and finally triggers apoptosis [40]. There is so a need to identify by which pathways calcium leaks from the ER of pancreatic beta cells submitted to lipotoxicity in order to find out drugs to restore ER calcium homeostasis. In addition, knowledge of modulation of ER calcium efflux during lipotoxicity would be useful for a better understanding of the ER calcium involvement in type 2 diabetes. This current study provides several important advances in this context.

We, and other groups, have demonstrated in several models that translocon is an ER calcium leak channel [24,25,27–31]. In addition, we have also shown that translocon is an ER calcium leak channel involved in ER calcium depletion in human cancerous prostatic cells in ER stress conditions [24]. This last study highlights the potential role of translocon in physiopathology. Translocon is an ubiquitous multiproteic complex also implied in protein translation and protein retro-translocation from the ER lumen to the cytosol, to be addressed to their target location. Accumulation of unfolded proteins leads to ER stress and UPR [17]. These unfolded proteins are retro-translocated from the ER lumen to the cytosol, through the translocon, and degraded by the proteasome [41,42]. ER stress is largely investigated currently in the literature and is shown to be central in a lot of cells integrated answers to glucolipotoxicity [43]. Furthermore, it is well established that under resting conditions, GRP78 (also called BiP) binds to the luminal part of ER membrane proteins like IRE1, PERK and ATF6, involved in UPR pathway (for review, see [11,17]). GRP78 is also known to seal the pore of the translocon [44] and so possibly inhibits ER calcium leak [32]. Accumulation of unfolded proteins induces ER stress, so GRP78 is released from IRE1α, PERK, ATF6 and then triggers the UPR response and probably enhances ER calcium depletion.

In the present work, we hypothesized that translocon could be one of the ER calcium leak channel involved in ER calcium depletion in pancreatic beta cells in physiologic and lipotoxic conditions. We found that its inhibition in lipotoxic conditions maintains beta cell calcium homeostasis and restores insulin secretion in human islets. Interestingly, a point mutation in mice was shown to be responsible for the development of type 2 diabetes [45]. Lloyd and coll. have demonstrated that insulin insufficiency was due to ER stress-induced apoptosis [46]. This interesting article highlights the fact that Sec61α, and so the translocon, appear to be involved in ER homeostasis in beta cells. Any perturbations of translocon either due to a mutation [47] or to pharmacological modulation (present work) may have important consequences in beta cell functions.

ER homeostasis is linked to regulation of its luminal calcium concentration, resulting from a right balance between intracellular calcium pumps and passive calcium leak channels. We demonstrate here that translocon acts as a functional ER calcium leak channel in pancreatic beta cells by measuring, in MIN6B1 cells and in human islets, a significant ER calcium depletion in response to acute perfusion of puromycin, which was abrogated by anisomycin. Anisomycin and puromycin did not modify the resting calcium concentration in the both models. Puromycin-induced ER calcium depletion in beta cells is specific to translocon as demonstrated in other cell types [24,30,31]. Indeed, previous inhibition of ryanodine receptors or IP_3_ receptors did not change either puromycin action on ER calcium content or its inhibition by anisomycin. These results highlight the involvement of translocon in ER calcium homeostasis as a functional reticular calcium leak channel in beta cells. In future studies, luminal and mitochondrial targeted calcium probes will help to precise the fine tuning that operates on translocon.

ER calcium homeostasis is involved in many cellular functions like ER stress and apoptosis in beta cells [46]. We first measured the long-term effects of palmitate on ER calcium content and the putative ability of anisomycin to maintain ER calcium homeostasis and beta cell function. Chronic palmitate treatment induced an ER calcium decrease and anisomycin partially protected ER calcium stores from depletion in human islets. We obtained similar results in murine pancreatic beta cells MIN6B1 treated by palmitate. GRP78 overexpression also did not change the resting calcium concentration in Min6B1 cells in control and in palmitate conditions. As expected, ER calcium content was enhanced in Min6B1 cells overexpressing GRP78 in control condition: GRP78 seals the pore of translocon and then reduces basal calcium leak. Nevertheless, this effect was not observed in chronic palmitate conditions. This may be due to a not enough effective GRP78 overexpression in our experimental conditions. To our knowledge, these data demonstrate for the first time that palmitate-induced toxicity may enhance ER calcium depletion via translocon. However we must noticed some limitations of the use of modulations of GRP78 expression. It was previously shown that overproduction of the ER chaperone GRP78 partially protects against the effects of palmitate in MIN6 cells [47] on ER stress effectors as Chop and XBP1.Therefore the overexpression of GRP78 could affect not only the activity of the translocon but will also improve ER homeostasis and attenuate ER stress. It would be very difficult to distinguish between the effects on the activity of the translocon and the general improvement of ER homeostasis.

To go further, we next measured the action of palmitate and anisomycin on SEC61α 1 expression. Recently, Cnop *et al*. [48] examined the transcriptome of human islets submitted to palmitate. Among all the genes studied, expression levels of transcripts for SEC61α 1 and GRP78 were enhanced, highlighting the importance of translocon in lipotoxic islets. In our study, we did not notice an enhancement of the amount of mRNA for SEC61α 1 whatever the experimental conditions. We hypothesized that anisomycin beneficial effects on ER calcium content is due to a direct action on its calcium activity and not its expression. Furthermore, we verified that anisomycin did not altered translation in a dose-dependent experiments. This result indicates that translocon is still functional for protein translation and could liberate calcium from the ER to the cytosol each time a protein is going through its pore.

We investigated the other arms of ER stress. Human islets were cultivated in mild palmitate chronic conditions to try to mimic first phase of type 2 diabetes [35,36,49]. Human islets showed an ER stress with mRNA overexpression of GRP78, known to allow UPR, and of CHOP and of XBP1s, ATF4 was not overexpressed under these conditions. These results on human islets, are in accordance with those from Boslem *et al*. showing in MIN6 cells that mild palmitate chronic treatment activated preferentially some ER stress pathways rather than others [50].

Our model of human islets treated with palmitate displayed an up-regulation of GRP78 and unspliced/spliced XBP1 with elevated levels of CHOP which is considered the activator factor of apoptotic pathways elicited by ER stress. In accordance with the fact that caspases were not activated by palmitate treatment in our conditions, we conclude that we have induced a mild ER stress in human islets as observed in the first phase of type 2 diabetes. We speculate that in the long term, calcium depletion and ER dysfunction could have a detrimental effect on beta cell survival. Indeed, a recent study has demonstrated that Ca^2+^ depletion induced by thapsigargin is associated with activation of ER stress, causing upregulation of both GRP78 and CHOP, eventually inducing cell death [51].

Anisomycin partially improves the reticular calcium concentration and was able to prevent palmitate-induced ER stress at GRP78 and CHOP mRNA levels. It is also interesting to notice that, however ER calcium is largely decreased, certainly participates to the moderate ER stress we observed, but is not sufficient to induce a caspase-dependent apoptosis. This suggest that ER calcium maintenance is crucial for the physiology of the cells but not necessary sufficient. Furthermore, recent studies suggested that whether calcium and ER function are tightly linked, there are possibilities for independent actions in pancreatic beta cells [50].

Chronic palmitate treatment is also known to inhibit glucose-induced insulin release [52]. As expected, we observed that mild chronic palmitate treatment reduces GSIS in human islets. We further observed in this study that anisomycin partly prevented ER calcium depletion. Therefore, we guess, in accordance with the literature, that restorations of ER calcium homeostasis should allow suitable insulin secretion in response to glucose and ER calcium liberation [43,53]. This was confirmed in human islets where beta cell function was protected from deleterious effects of lipotoxicity in presence of anisomycin. Anisomycin inhibition of ER calcium depletion suggest that beta cell dysfunction due to lipotoxicity should be mainly triggered by ER calcium depletion rather than an increase in cytosolic calcium concentration. These results are in accordance with data showing that SERCA mRNA expression was not affected by FFAs and that there was neither ER stress induction nor impairment of glucose tolerance in SERCA3−/− mice indicating that SERCA3 might not be a culprit in the etiology of T2D as previously suggested (see[52] for review). Indeed, we did not notice any lasting increase in cytosolic calcium concentration in cells treated with palmitate and anisomycin. However, cytosolic calcium increase due to increased ER calcium leak in lipotoxic conditions [23] could have effects on beta cell function as GSIS. Anisomycin, by translocon inhibition could also act on this phenomenon.

Interestingly, translocon inhibition restores ER calcium concentration improvement and insulin secretion in human islets submitted to lipotoxic conditions without affecting all the ways of ER stress. This is an original case of partial dissociation of ER calcium homeostasis and ER function that could help to better understand how calcium regulation by the ER preserves insulin secretion in pancreatic beta cells.

**Fig 5:**
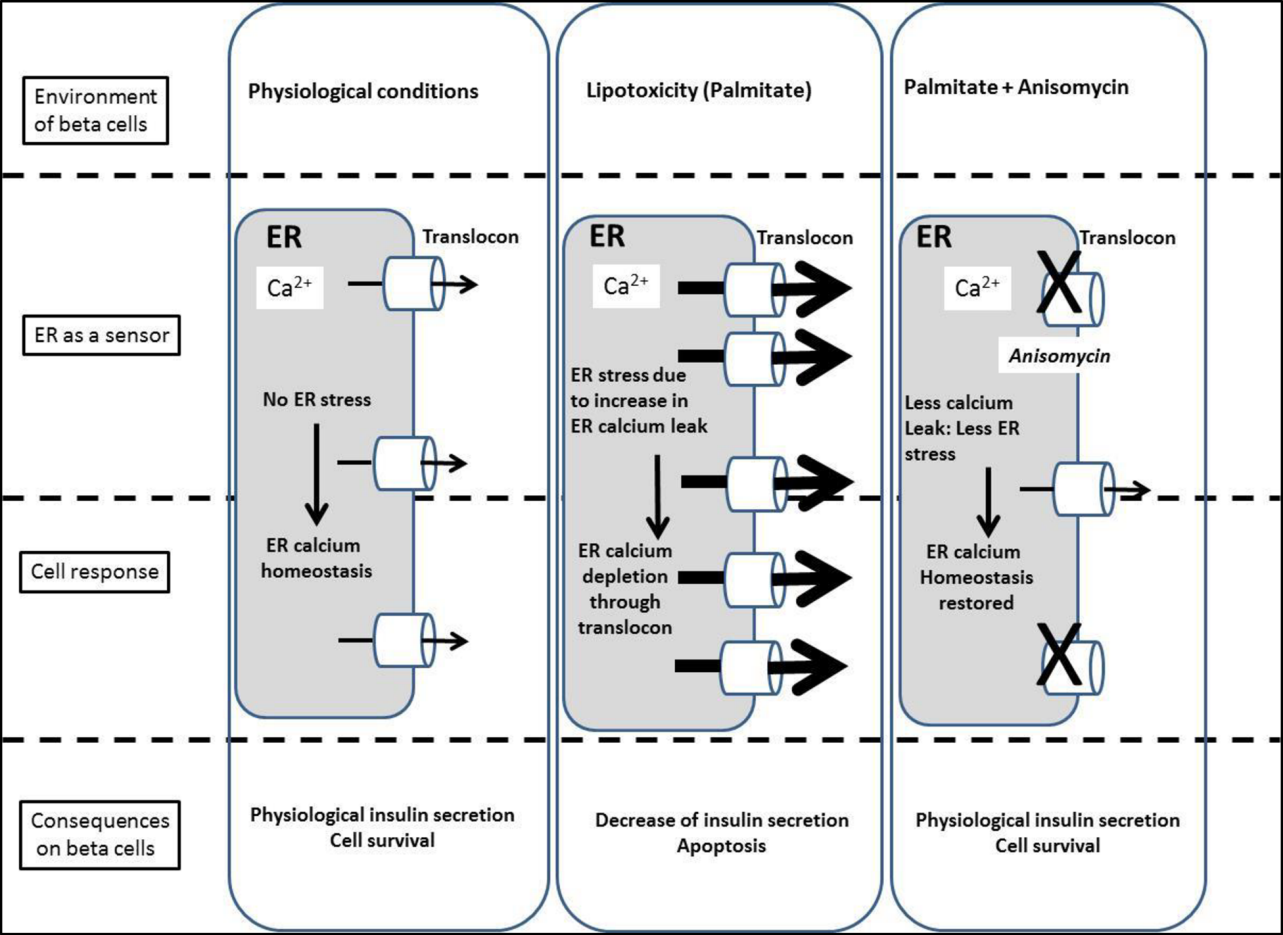
Model of translocon implication in pancreatic beta cells. Chronic palmitate causes ER calcium depletion due to a greater ER calcium leak through the translocon, triggering ER stress and decreased glucose-stimulated insulin release, indicating an altered metabolism-secretion coupling. Addition of anisomycin reduced ER stress and ER calcium leak from translocon, leading to restored insulin secretion.

To our knowledge, this is the first study to show that the translocon is implicated in both the global calcium homeostasis in pancreatic beta cells, and in lipotoxicity-induced beta cell dysfunction. Its inhibition resulted in improved calcium homeostasis, ER stress and insulino-secretion in human islets as illustrated (Fig 5).

Taken together, these results suggest that the control of calcium homeostasis by translocon inhibition could be a new strategy for preventing and treating type 2 diabetes as a part of combinatorial approaches that might be required to preserve or restore beta cell function as recently concluded in Concensus Statement on type 2 diabetes [54].

## Supporting Information

S1 Fig Anisomycin dose-responses in MIN6B1 cells. Quantitative analysis of protein expression. (a) Western blot from a representative experiment. (b) Analysis of protein expression (n = 3). MIN6B1 were cultured in control conditions during 24h without or with increasing anisomycin concentrations (0.1 to 2 μM; shading grey bars). ** p<0.01.
(PPTX)

S2 Fig Puromycin and anisomycin acute effects on calcium homeostasis in MIN6B1 cells. Puromycin and anisomcyin effects were evaluated in 4 different preparations of MIN6B1 (51 to 82 different cells) (**a**-**e**). Typical cytosolic calcium traces in response to 200 μM puromycin (**a**) or to 1 μM thapsigargin (**b**) under control conditions and after 30 min incubation with 200 μM anisomycin. Cumulative data of peak cytosolic calcium increases evoked by puromycin responses under control conditions and with anisomycin 200 μM (**c**). Cumulative data of peak cytosolic calcium increases evoked by thapsigargin responses (**d**) and (**e**) resting fluorescence (F340/F380) under control conditions ± puromycin or anisomycin pretreatment. *p<0.05, **p<0.01. Measures were assessed in a calcium-free medium. Preparations were done in duplicate.
(PPTX)

S3 Fig Puromycin and anisomycin modulate specifically calcium release from translocon in MIN6B1 cells. (white bar) Puromycin (200μM) induced an ER calcium release (n = 12). (black bar) Anisomycin (200μM) inhibited puromycin induced calcium release when added 1h before puromycin (n = 14). (dark grey bar) Puromycin (200μM) induced a calcium release in presence of ryanodine (50μM) added 1h before puromycin (n = 8). (light grey bar) Puromycin (200μM) induced an ER calcium release after treatment with xestospongin C (3μM) (n = 8). Data were obtained from 3 different experiments. Statistical analysis were done with an Anova test *p<0.05, **p<0.01, ***p<0.001.
(PPTX)

S4 Fig Dynamic changes in ER Ca^2+^ in MIN6B1 cells, under resting conditions and after stimulation with thapsigargin (TG; 1 μM), were monitored after a 20 min pre-treatment with or without anisomycin (200 μM) using Ad-D4ER. Anisomycin was also present during the measurement period. **(a)** Average traces (black: control, grey: anisomycin) of ER Ca^2+^ (R/R_0_) are shown as FRET ratio (R:YCFP/CFP) after correction of background/photobleaching and normalization with the basal FRET ratio (R_0_) (n = 90 & 99 cells for control and anisomycin respectively). **(b)** Analysis of the statistical differences (Δ_max_) in ER Ca^2+^ before and after TG treatment reflecting the amount of Ca^2+^ released from ER. **(c)** Resting ER Ca^2+^ data extracted from the basal FRET ratio before normalization with R_0_. The bars in panels (**b**) and (**c**) represent mean ratio ± SEM. ***p = 0.0001, *p = 0.02 for anisomycin vs control.
(TIF)

S5 Fig Chronic effects of palmitate and anisomycin treatments on calcium homeostasis in MIN6B1 cells. Quantification of fluorescence ratio (F340/F380) of resting calcium (**a**) and of reticular calcium release (**b**) induced by 1 μM thapsigargin in MIN6B1 cells under BSA (white bar) + anisomycin (grey bars) and palmitate (black bar) conditions + anisomycin (hatched bars). *p<0.05, **p<0.01. N = 33–44 at least from 3 independent MIN6 cultures; preparations in duplicate.
(PPTX)

S6 Fig Cytosolic Ca^2+^ measured in MIN6B1 cells upon GRP78 overexpression using Fura-2 AM. (**a**) Typical cytosolic calcium traces in response to 1 μM thapsigargin. Cumulative data of (**b**) resting fluorescence (F340/F380) and (**c**) peak cytosolic calcium increases evoked by thapsigargin responses under control conditions (BSA) or palmitate pretreatment. *p<0.05, **p<0.01, ***p<0.001. Measures were assessed in a calcium-free medium. Preparations were done in triplicate (n = 55–66 cells).
(PPTX)

S7 Fig GRP-78 adenovirus dose-responses in MIN6B1 cells. Quantitative analysis of protein expression. (**a**) Western blot from a representative experiment. (**b**) Analysis of protein expression (n = 3). MIN6B1 were cultured in control conditions during 48h without or with increasing GRP78-adenovirus concentrations (0.1 to 2. μl). *** p<0.001; ** p<0.01; * p<0.05.
(PPTX)

S8 Fig GRP-78 protein knock-down in MIN6B1 cells. Quantitative analysis of protein expression. (**a**) Western blot from a representative experiment. (**b**) Analysis of protein expression (n = 3). MIN6B1 were cultured in control conditions during 48h without or with GRP78 SiRNAs. ** p<0.01.
(PPTX)

S1 Table Primer sequences used for qRT-PCR. (DOCX)
