# Supplementary material for: Protection of Human Pancreatic Islets from Lipotoxicity by Modulation of the Translocon
Source: PLoS One. 2016 Feb 10;11(2):e0148686. doi: 10.1371/journal.pone.0148686 (PMC4749224; doi:10.1371/journal.pone.0148686)
Supplement: S1 Fig — Quantitative analysis of protein expression. (a) Western blot from a representative experiment. (b) Analysis of protein expression (n = 3). MIN6B1 were cultured in control conditions during 24h without or with increasing anisomycin concentrations (0.1 to 2 μM; shading grey bars). ** p<0.01. (PPTX) [file pone.0148686.s001.pptx]

## Slide 1
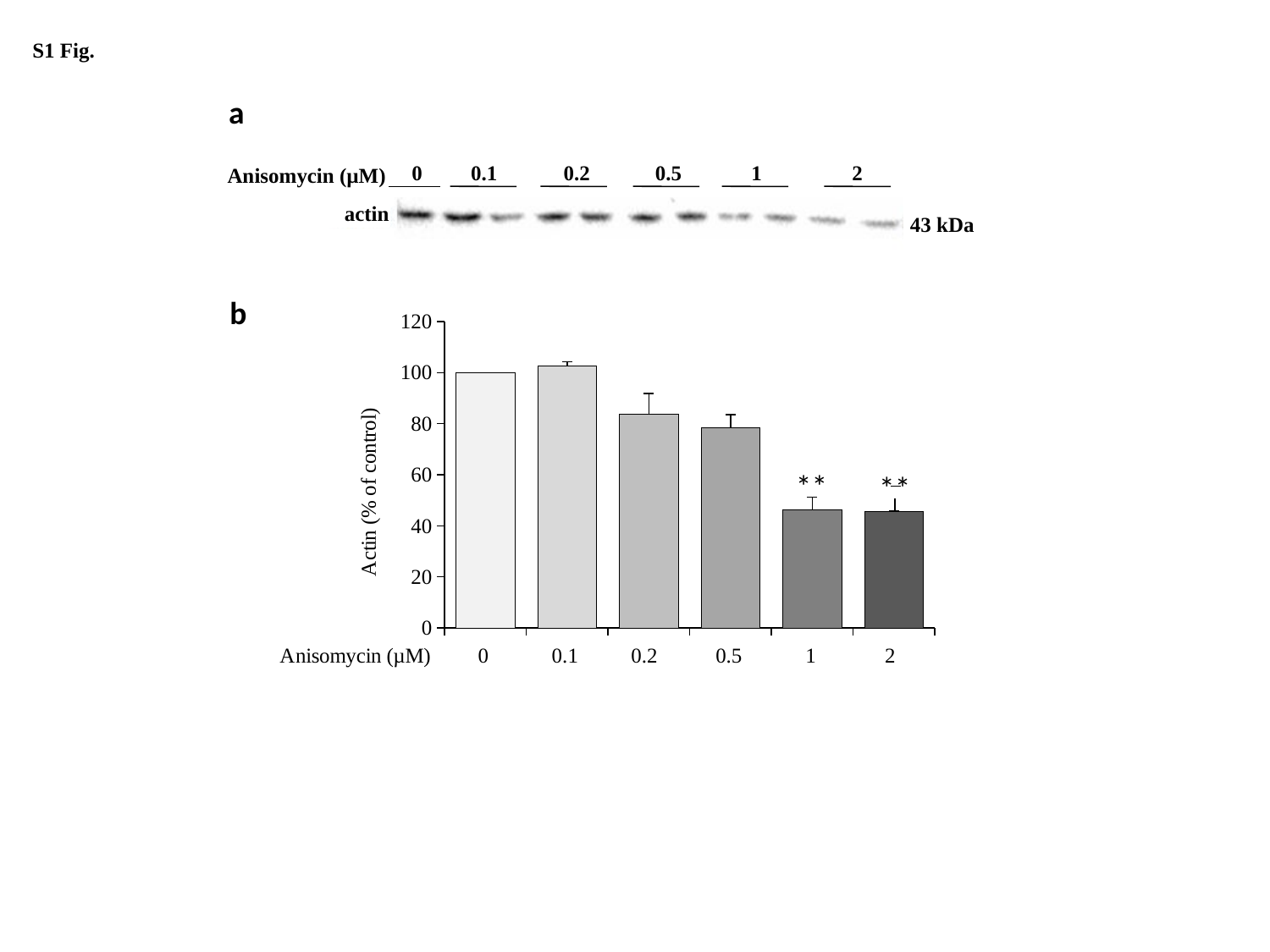

S1 Fig.
a
0
0.1
0.2
0.5
1
2
Anisomycin (µM)
actin
43 kDa
b
### Chart
| Category | |
|---|---|
| BSA | 100.0 |
| BSA + Ao 100 nM | 102.4905477886222 |
| BSA + Ao 200 nM | 83.74219643014158 |
| BSA + Ao 500 nM | 78.50171458718016 |
| BSA + Ao 1 uM | 46.087224127319 |
| BSA + Ao 2 uM | 45.55965884111492 |**
**
