# Supplementary material for: Protection of Human Pancreatic Islets from Lipotoxicity by Modulation of the Translocon
Source: PLoS One. 2016 Feb 10;11(2):e0148686. doi: 10.1371/journal.pone.0148686 (PMC4749224; doi:10.1371/journal.pone.0148686)
Supplement: S2 Fig — Puromycin and anisomcyin effects were evaluated in 4 different preparations of MIN6B1 (51 to 82 different cells) (a-e). Typical cytosolic calcium traces in response to 200 μM puromycin (a) or to 1 μM thapsigargin (b) under control conditions and after 30 min incubation with 200 μM anisomycin. Cumulative data of peak cytosolic calcium increases evoked by puromycin responses under control conditions and with anisomycin 200 μM (c). Cumulative data of peak cytosolic calcium increases evoked by thapsigargin responses (d) and (e) resting fluorescence (F340/F380) under control conditions ± puromycin or anisomycin pretreatment. *p<0.05, **p<0.01. Measures were assessed in a calcium-free medium. Preparations were done in duplicate. (PPTX) [file pone.0148686.s002.pptx]

## Slide 1
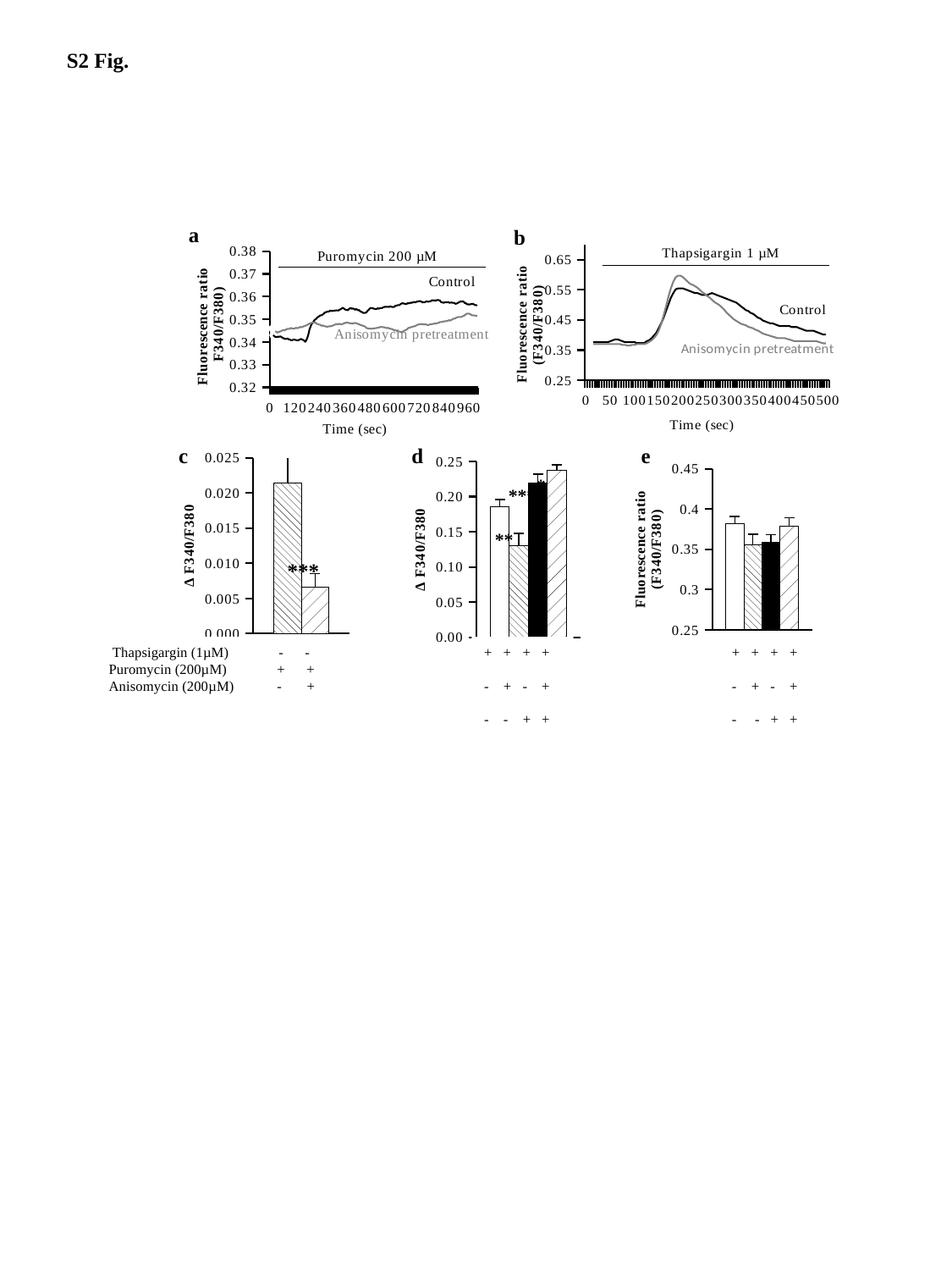

S2 Fig.
a
b
### Chart
| Category | | |
|---|---|---|
| 0.0 | 0.343702862877822 | 0.344847777382313 |
| 5.0 | 0.343459793255926 | 0.346628803074328 |
| 10.0 | 0.342240100917565 | 0.344230941597845 |
| 15.0 | 0.342410696647493 | 0.343922517878669 |
| 20.0 | 0.342251621872104 | 0.3446381812382 |
| 25.0 | 0.341942079694639 | 0.343894521036898 |
| 30.0 | 0.342112992137407 | 0.343510915914363 |
| 35.0 | 0.342802710098099 | 0.344445324161613 |
| 40.0 | 0.342293870977103 | 0.34572833328372 |
| 45.0 | 0.34204034904318 | 0.344632913714079 |
| 50.0 | 0.34275848450024 | 0.345796420901933 |
| 55.0 | 0.341489553105049 | 0.344592775735908 |
| 60.0 | 0.34116219808159 | 0.346001805926301 |
| 65.0 | 0.34106704604541 | 0.34461379625298 |
| 70.0 | 0.341850123864575 | 0.344614487430779 |
| 75.0 | 0.341094406605643 | 0.347311072661672 |
| 80.0 | 0.341628050106563 | 0.346065467542727 |
| 85.0 | 0.341390038735041 | 0.346227840832189 |
| 90.0 | 0.340549733314113 | 0.345484247232659 |
| 95.0 | 0.34058918296893 | 0.345312506435144 |
| 100.0 | 0.340783981211999 | 0.346848398347582 |
| 105.0 | 0.340988264062207 | 0.345781614115441 |
| 110.0 | 0.34135399389229 | 0.345857331227067 |
| 115.0 | 0.34113974184586 | 0.345755285423583 |
| 120.0 | 0.340232773787892 | 0.346429523751013 |
| 125.0 | 0.34061134142304 | 0.346838015295367 |
| 130.0 | 0.340927721370904 | 0.345670268468206 |
| 135.0 | 0.341069380891205 | 0.346498461497103 |
| 140.0 | 0.34109831525502 | 0.346629671680315 |
| 145.0 | 0.341613606723663 | 0.347488267551439 |
| 150.0 | 0.341414177218845 | 0.346318789079428 |
| 155.0 | 0.340166551386247 | 0.346545584811519 |
| 160.0 | 0.340184779627251 | 0.347509027664166 |
| 165.0 | 0.340335809785565 | 0.34708299211617 |
| 170.0 | 0.339601424762778 | 0.348019807216116 |
| 175.0 | 0.343031780408313 | 0.348130759921138 |
| 180.0 | 0.344550343249428 | 0.348081525488693 |
| 185.0 | 0.346582670606811 | 0.348118125048786 |
| 190.0 | 0.347672123398252 | 0.34893131630155 |
| 195.0 | 0.347782164936428 | 0.34830364979259 |
| 200.0 | 0.349509882788947 | 0.348987502940392 |
| 205.0 | 0.348644136867611 | 0.348715452410315 |
| 210.0 | 0.3502107793035 | 0.348114559530207 |
| 215.0 | 0.350536748875262 | 0.348516503812128 |
| 220.0 | 0.350490691786432 | 0.348158115158783 |
| 225.0 | 0.350635489250194 | 0.345530199416982 |
| 230.0 | 0.352075707254312 | 0.348121227750417 |
| 235.0 | 0.351676911544228 | 0.347692394925804 |
| 240.0 | 0.351802997569538 | 0.347740748480607 |
| 245.0 | 0.351552892264281 | 0.347201494449993 |
| 250.0 | 0.352016007532957 | 0.346103909901292 |
| 255.0 | 0.353460078632996 | 0.346716097443027 |
| 260.0 | 0.353065849227975 | 0.346836408922407 |
| 265.0 | 0.353491929489222 | 0.346643777575042 |
| 270.0 | 0.35232520448349 | 0.347585432442081 |
| 275.0 | 0.354498322915079 | 0.345938863379949 |
| 280.0 | 0.353253303964758 | 0.347041930641045 |
| 285.0 | 0.35377062635975 | 0.347589300961014 |
| 290.0 | 0.353813628824881 | 0.346203966769884 |
| 295.0 | 0.3536861850906 | 0.348082862409023 |
| 300.0 | 0.353404425210289 | 0.347525240937589 |
| 305.0 | 0.353932971152527 | 0.347472614843645 |
| 310.0 | 0.354021448128993 | 0.348214241845486 |
| 315.0 | 0.354072735311157 | 0.348703372817908 |
| 320.0 | 0.353664889966601 | 0.347342485732404 |
| 325.0 | 0.353722916825494 | 0.347439780161097 |
| 330.0 | 0.353921983652556 | 0.34789490278488 |
| 335.0 | 0.355550400600518 | 0.34835703585164 |
| 340.0 | 0.354331307804021 | 0.34714025439581 |
| 345.0 | 0.354777490763594 | 0.348623115161558 |
| 350.0 | 0.355480663576931 | 0.348178341124296 |
| 355.0 | 0.354754398725939 | 0.348497268019783 |
| 360.0 | 0.352513758409601 | 0.349502647424508 |
| 365.0 | 0.354219105920381 | 0.34775315476146 |
| 370.0 | 0.354688595487621 | 0.349450208633469 |
| 375.0 | 0.354503675405286 | 0.347377420215526 |
| 380.0 | 0.354349646667379 | 0.347498664933838 |
| 385.0 | 0.355544430825801 | 0.348424265098878 |
| 390.0 | 0.355198908601081 | 0.347846633083932 |
| 395.0 | 0.35332098802941 | 0.349059776536313 |
| 400.0 | 0.354953260087833 | 0.347890516125692 |
| 405.0 | 0.355041192077215 | 0.349303355405767 |
| 410.0 | 0.353230147148243 | 0.347607816395366 |
| 415.0 | 0.354849267563983 | 0.347689730379779 |
| 420.0 | 0.354084907096009 | 0.347735280558957 |
| 425.0 | 0.353494424749265 | 0.346936901506133 |
| 430.0 | 0.353789642862545 | 0.347703716101559 |
| 435.0 | 0.352721223429167 | 0.34733934250008 |
| 440.0 | 0.353170409042143 | 0.346908351164121 |
| 445.0 | 0.352609162382824 | 0.347053934784379 |
| 450.0 | 0.352656457642767 | 0.34599288679453 |
| 455.0 | 0.352690844548055 | 0.34700323312092 |
| 460.0 | 0.353396735003797 | 0.34538022666269 |
| 465.0 | 0.353425580267863 | 0.344394198917262 |
| 470.0 | 0.355079330399964 | 0.34648921247872 |
| 475.0 | 0.354426691585933 | 0.345932736872983 |
| 480.0 | 0.35530114243822 | 0.346331173063888 |
| 485.0 | 0.355134606281779 | 0.346188878706922 |
| 490.0 | 0.354622577305462 | 0.345558830268378 |
| 495.0 | 0.354628580134065 | 0.345261069956837 |
| 500.0 | 0.35425588105425 | 0.346338306202713 |
| 505.0 | 0.354852763635255 | 0.346638963864372 |
| 510.0 | 0.354403194387375 | 0.34638016638841 |
| 515.0 | 0.354749614815493 | 0.346653432725804 |
| 520.0 | 0.355395251930576 | 0.346494887332747 |
| 525.0 | 0.354066631561274 | 0.345685921346929 |
| 530.0 | 0.355154676479138 | 0.347344487041537 |
| 535.0 | 0.355182573693716 | 0.347091306671417 |
| 540.0 | 0.355181168285037 | 0.346099706179089 |
| 545.0 | 0.355345809919001 | 0.346456986936606 |
| 550.0 | 0.355888324211624 | 0.346043395905585 |
| 555.0 | 0.355463339622352 | 0.345200072733435 |
| 560.0 | 0.355051814303408 | 0.346295970388675 |
| 565.0 | 0.355568162671364 | 0.346452052499302 |
| 570.0 | 0.355762351354665 | 0.346981927240264 |
| 575.0 | 0.355287168745876 | 0.344444385453813 |
| 580.0 | 0.356175883256528 | 0.345719941460711 |
| 585.0 | 0.354832395055685 | 0.344610693041915 |
| 590.0 | 0.355375709682369 | 0.345620938357874 |
| 595.0 | 0.355307854503075 | 0.344850400298025 |
| 600.0 | 0.356318975472308 | 0.345057934038581 |
| 605.0 | 0.356665833012697 | 0.345444503708729 |
| 610.0 | 0.355614903631693 | 0.344223572348648 |
| 615.0 | 0.356081218391158 | 0.34393876894304 |
| 620.0 | 0.356650213800105 | 0.34393308309924 |
| 625.0 | 0.356653377826415 | 0.344912142616191 |
| 630.0 | 0.357311091720591 | 0.344103341791677 |
| 635.0 | 0.357197319370756 | 0.344548281864083 |
| 640.0 | 0.357054317205708 | 0.345559831295454 |
| 645.0 | 0.355962985735367 | 0.346144161233983 |
| 650.0 | 0.357114865595133 | 0.345122722943332 |
| 655.0 | 0.356880766030876 | 0.345994102524695 |
| 660.0 | 0.357192288733479 | 0.345728244918437 |
| 665.0 | 0.357391231380798 | 0.347094847557104 |
| 670.0 | 0.356491990141713 | 0.347591754981675 |
| 675.0 | 0.357419004559858 | 0.346141133079281 |
| 680.0 | 0.358050947830658 | 0.346729493699346 |
| 685.0 | 0.357021923664593 | 0.346450538939687 |
| 690.0 | 0.357238385288805 | 0.346593375597932 |
| 695.0 | 0.358084738558556 | 0.347810319010066 |
| 700.0 | 0.35715665395129 | 0.347998769316251 |
| 705.0 | 0.35810684092143 | 0.348214045083878 |
| 710.0 | 0.357733208002599 | 0.347462765911481 |
| 715.0 | 0.35847296458873 | 0.34786534906191 |
| 720.0 | 0.357647273401756 | 0.347422186959031 |
| 725.0 | 0.357897421594149 | 0.347645632538305 |
| 730.0 | 0.357153431387696 | 0.348963901469889 |
| 735.0 | 0.35696672443406 | 0.347357463247714 |
| 740.0 | 0.357973979678632 | 0.347526008433015 |
| 745.0 | 0.357794310654368 | 0.347229610888907 |
| 750.0 | 0.357820732537277 | 0.347846166786138 |
| 755.0 | 0.357934103733478 | 0.347214321819109 |
| 760.0 | 0.35742836189594 | 0.347525948204445 |
| 765.0 | 0.357570250297605 | 0.347532961120111 |
| 770.0 | 0.358726490763537 | 0.348824308658637 |
| 775.0 | 0.35802571570207 | 0.34763224767436 |
| 780.0 | 0.358893372876619 | 0.348399675052028 |
| 785.0 | 0.357785355687435 | 0.347588948362143 |
| 790.0 | 0.358173497543055 | 0.348014126014018 |
| 795.0 | 0.358578162771958 | 0.348408499095841 |
| 800.0 | 0.357847616173808 | 0.348575137121728 |
| 805.0 | 0.359247669359851 | 0.348312561456085 |
| 810.0 | 0.358357607735577 | 0.34914295542019 |
| 815.0 | 0.358068978374348 | 0.348724936522581 |
| 820.0 | 0.357144419811248 | 0.3492958977717 |
| 825.0 | 0.356749712242361 | 0.348574970749847 |
| 830.0 | 0.357723681161659 | 0.349061790910814 |
| 835.0 | 0.357648910598169 | 0.348714071653204 |
| 840.0 | 0.357010116162782 | 0.350238927747961 |
| 845.0 | 0.357845644771106 | 0.349149837577282 |
| 850.0 | 0.35722181646928 | 0.349221011985667 |
| 855.0 | 0.357646433909064 | 0.349252593207003 |
| 860.0 | 0.357613170371283 | 0.349929064713614 |
| 865.0 | 0.356541487135295 | 0.34978688271903 |
| 870.0 | 0.357335564053537 | 0.349160849559857 |
| 875.0 | 0.358070343731189 | 0.350515274274474 |
| 880.0 | 0.356954965357968 | 0.351212160192836 |
| 885.0 | 0.356790639185376 | 0.350718353035464 |
| 890.0 | 0.356625492661831 | 0.350323482103159 |
| 895.0 | 0.356806907017383 | 0.351167671757988 |
| 900.0 | 0.357406554983478 | 0.35065790836056 |
| 905.0 | 0.357652210753351 | 0.351371470084727 |
| 910.0 | 0.357633533982371 | 0.351874211859469 |
| 915.0 | 0.357834460346132 | 0.350427735348819 |
| 920.0 | 0.358227890001697 | 0.35045649230527 |
| 925.0 | 0.357285224981142 | 0.351419319143576 |
| 930.0 | 0.358225535227115 | 0.352338504988525 |
| 935.0 | 0.35690673439946 | 0.353042680704435 |
| 940.0 | 0.356198501241303 | 0.352764479338179 |
| 945.0 | 0.357110718717921 | 0.3534751560986 |
| 950.0 | 0.356073988563041 | 0.351416708695321 |
| 955.0 | 0.356834017995371 | 0.352109397654199 |
| 960.0 | 0.356144643757544 | 0.35186023580793 |
| 965.0 | 0.356966454358943 | 0.352078049742752 |
| 970.0 | 0.357082500882321 | 0.35120489666684 |
| 975.0 | 0.35647522532862 | 0.351491222116209 |
| 980.0 | 0.356816236179291 | 0.351846669687359 |
| 985.0 | 0.35533754099611 | 0.351614410181461 |
| 990.0 | 0.356418836140888 | 0.351165030030151 |
| 995.0 | 0.356201030297472 | 0.351975384502888 |
| 1000.0 | 0.356454167496133 | 0.351579083019378 |
[unsupported chart]
c
d
e
### Chart
| Category | | |
|---|---|---|
### Chart
| Category | | | | |
|---|---|---|---|---|
### Chart
| Category | | | | |
|---|---|---|---|---| Thapsigargin (1µM) - -
 Puromycin (200µM) + +
 Anisomycin (200µM) - +
+ + + +
- + - +
- - + +
+ + + +
- + - +
- - + +
