# Supplementary figures and images for: Protection of Human Pancreatic Islets from Lipotoxicity by Modulation of the Translocon
Source: PLoS One. 2016 Feb 10;11(2):e0148686. doi: 10.1371/journal.pone.0148686 (PMC4749224; doi:10.1371/journal.pone.0148686)

## Slide 1
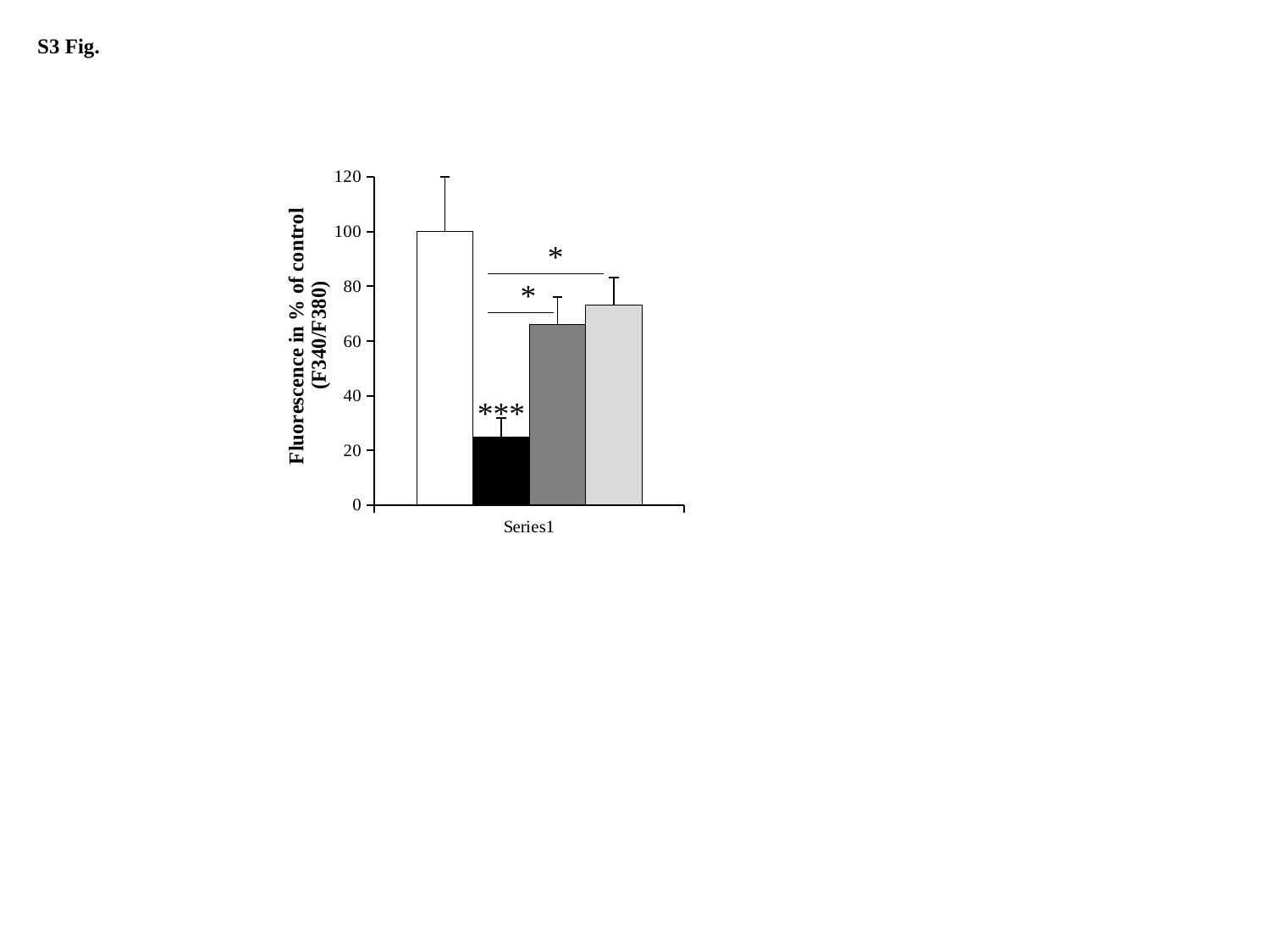

S3 Fig.
### Chart
| Category | | | | |
|---|---|---|---|---|
| | 100.0 | 24.77999999999999 | 66.1 | 73.03 |

Supplement: S3 Fig — (white bar) Puromycin (200μM) induced an ER calcium release (n = 12). (black bar) Anisomycin (200μM) inhibited puromycin induced calcium release when added 1h before puromycin (n = 14). (dark grey bar) Puromycin (200μM) induced a calcium release in presence of ryanodine (50μM) added 1h before puromycin (n = 8). (light grey bar) Puromycin (200μM) induced an ER calcium release after treatment with xestospongin C (3μM) (n = 8). Data were obtained from 3 different experiments. Statistical analysis were done with an Anova test *p<0.05, **p<0.01, ***p<0.001. (PPTX) [file pone.0148686.s003.pptx]

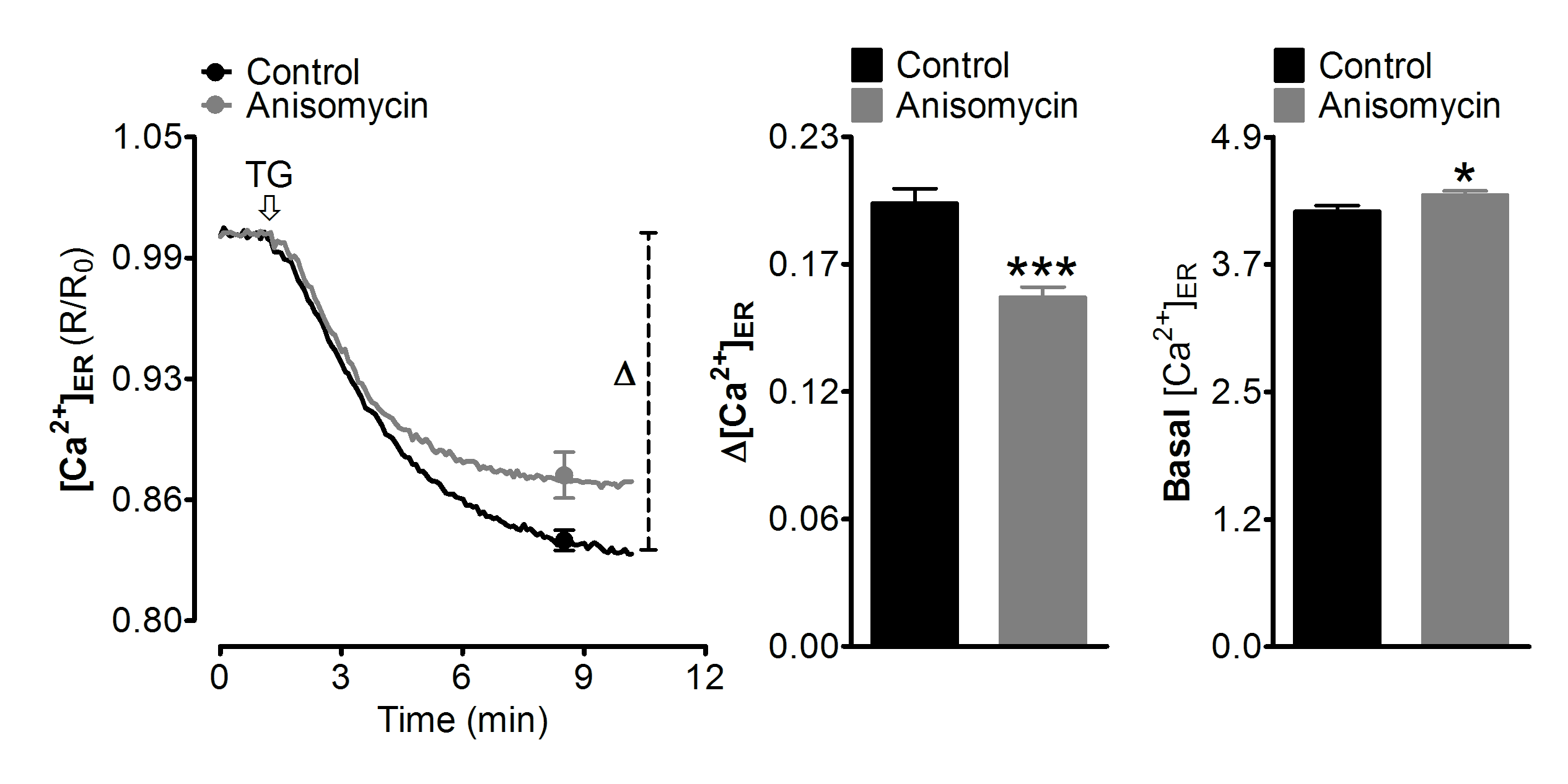

Supplement: S4 Fig — Anisomycin was also present during the measurement period. (a) Average traces (black: control, grey: anisomycin) of ER Ca2+ (R/R0) are shown as FRET ratio (R:YCFP/CFP) after correction of background/photobleaching and normalization with the basal FRET ratio (R0) (n = 90 & 99 cells for control and anisomycin respectively). (b) Analysis of the statistical differences (Δmax) in ER Ca2+ before and after TG treatment reflecting the amount of Ca2+ released from ER. (c) Resting ER Ca2+ data extracted from the basal FRET ratio before normalization with R0. The bars in panels (b) and (c) represent mean ratio ± SEM. ***p = 0.0001, *p = 0.02 for anisomycin vs control. (TIF) [file pone.0148686.s004.tif]

## Slide 1
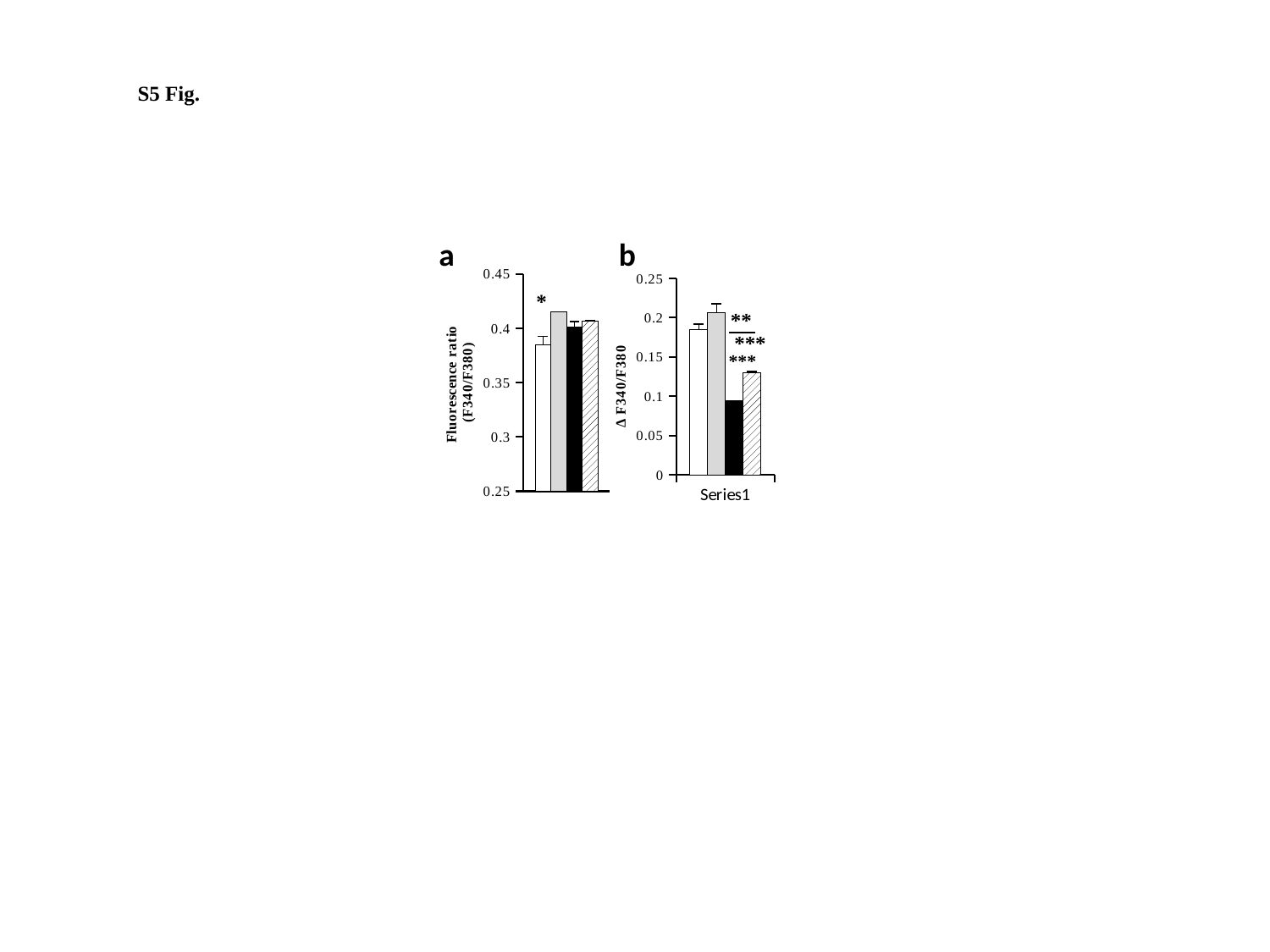

S5 Fig.
a
b
### Chart
| Category | | | | |
|---|---|---|---|---|
| BSA vs BSA AO | 0.384832587261001 | 0.414741852002764 | 0.40117914809456 | 0.406669807197355 |
### Chart
| Category | | | | |
|---|---|---|---|---|
| | 0.184472023864419 | 0.206454398059665 | 0.09392127478962 | 0.130209311329124 |

Supplement: S5 Fig — Quantification of fluorescence ratio (F340/F380) of resting calcium (a) and of reticular calcium release (b) induced by 1 μM thapsigargin in MIN6B1 cells under BSA (white bar) + anisomycin (grey bars) and palmitate (black bar) conditions + anisomycin (hatched bars). *p<0.05, **p<0.01. N = 33–44 at least from 3 independent MIN6 cultures; preparations in duplicate. (PPTX) [file pone.0148686.s005.pptx]

## Slide 1
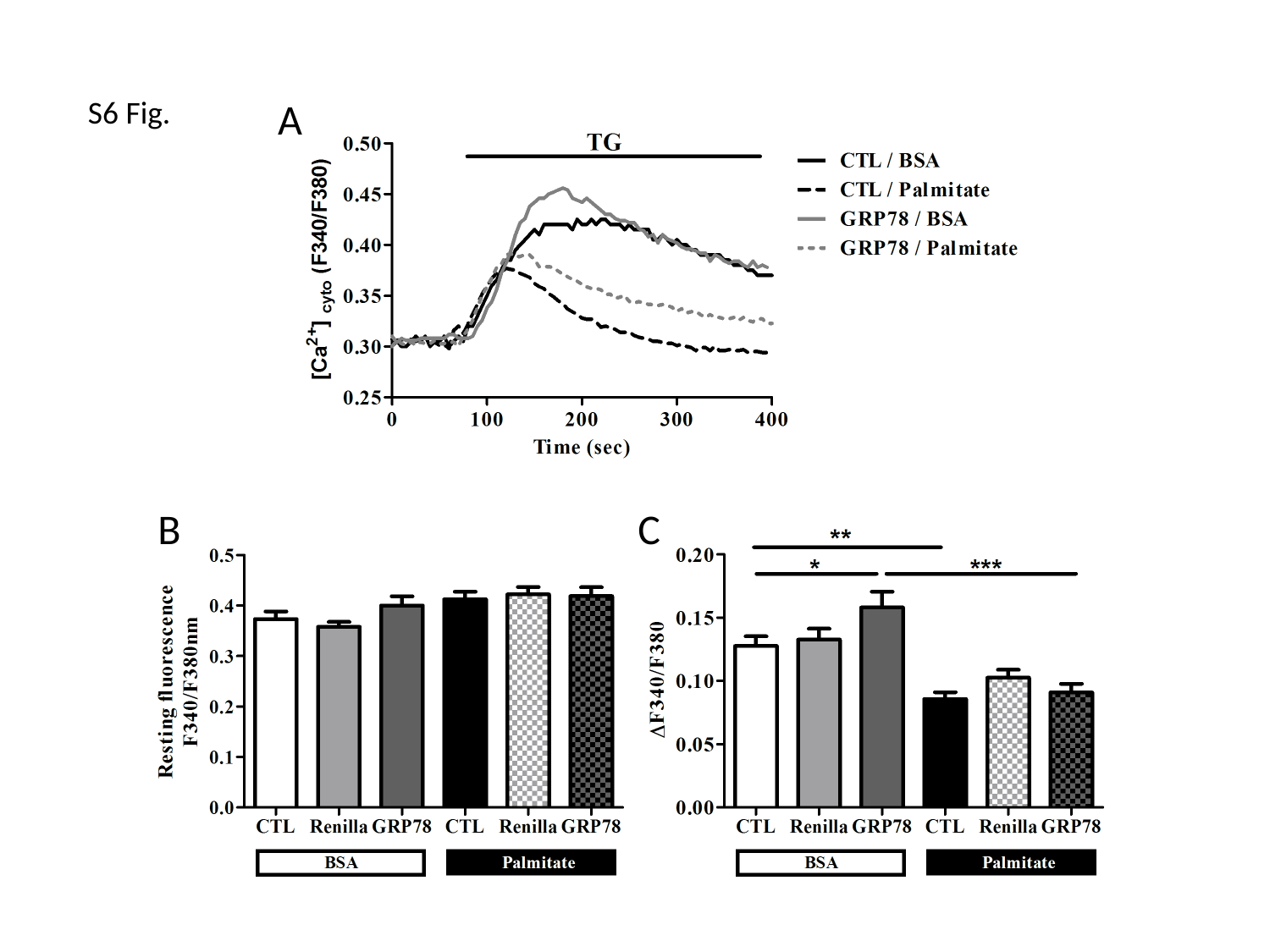

S6 Fig.
A
B
C

Supplement: S6 Fig — (a) Typical cytosolic calcium traces in response to 1 μM thapsigargin. Cumulative data of (b) resting fluorescence (F340/F380) and (c) peak cytosolic calcium increases evoked by thapsigargin responses under control conditions (BSA) or palmitate pretreatment. *p<0.05, **p<0.01, ***p<0.001. Measures were assessed in a calcium-free medium. Preparations were done in triplicate (n = 55–66 cells). (PPTX) [file pone.0148686.s006.pptx]

## Slide 1
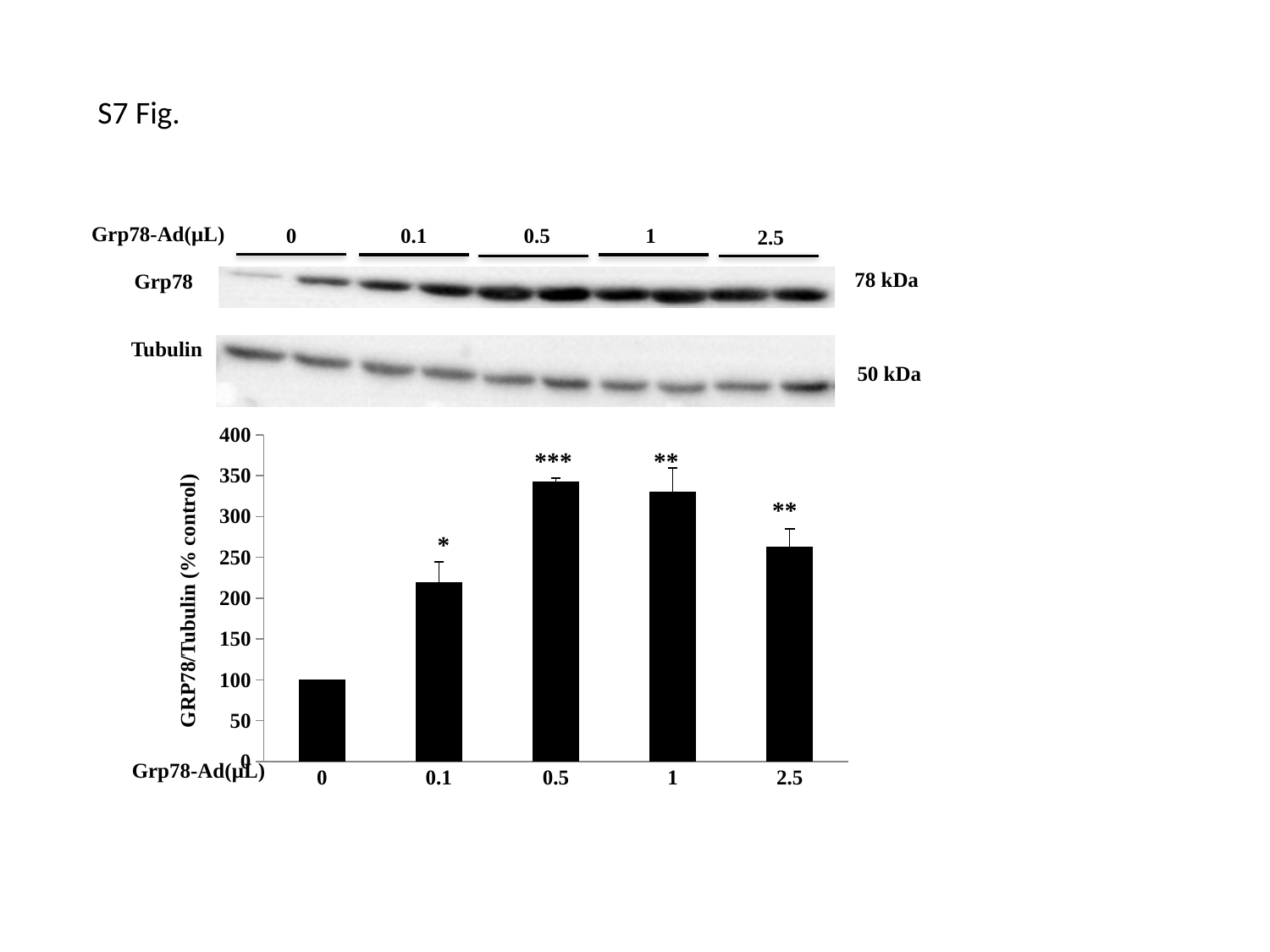

S7 Fig.
Grp78-Ad(μL)
0
0.5
0.1
2.5
78 kDa
Grp78
Tubulin
50 kDa
1
0,5
### Chart
| Category | |
|---|---|
| 0 | 100.0 |
| 0.1 | 219.98090720199357 |
| 0.5 | 342.7943967556048 |
| 1 | 330.0806159384082 |
| 2.5 | 262.7735084387472 |GRP78/Tubulin (% control)
Grp78-Ad(μL)
***
**
**
*

Supplement: S7 Fig — Quantitative analysis of protein expression. (a) Western blot from a representative experiment. (b) Analysis of protein expression (n = 3). MIN6B1 were cultured in control conditions during 48h without or with increasing GRP78-adenovirus concentrations (0.1 to 2. μl). *** p<0.001; ** p<0.01; * p<0.05. (PPTX) [file pone.0148686.s007.pptx]
