# Supplementary material for: Protection of Human Pancreatic Islets from Lipotoxicity by Modulation of the Translocon
Source: PLoS One. 2016 Feb 10;11(2):e0148686. doi: 10.1371/journal.pone.0148686 (PMC4749224; doi:10.1371/journal.pone.0148686)
Supplement: S8 Fig — Quantitative analysis of protein expression. (a) Western blot from a representative experiment. (b) Analysis of protein expression (n = 3). MIN6B1 were cultured in control conditions during 48h without or with GRP78 SiRNAs. ** p<0.01. (PPTX) [file pone.0148686.s008.pptx]

## Slide 1
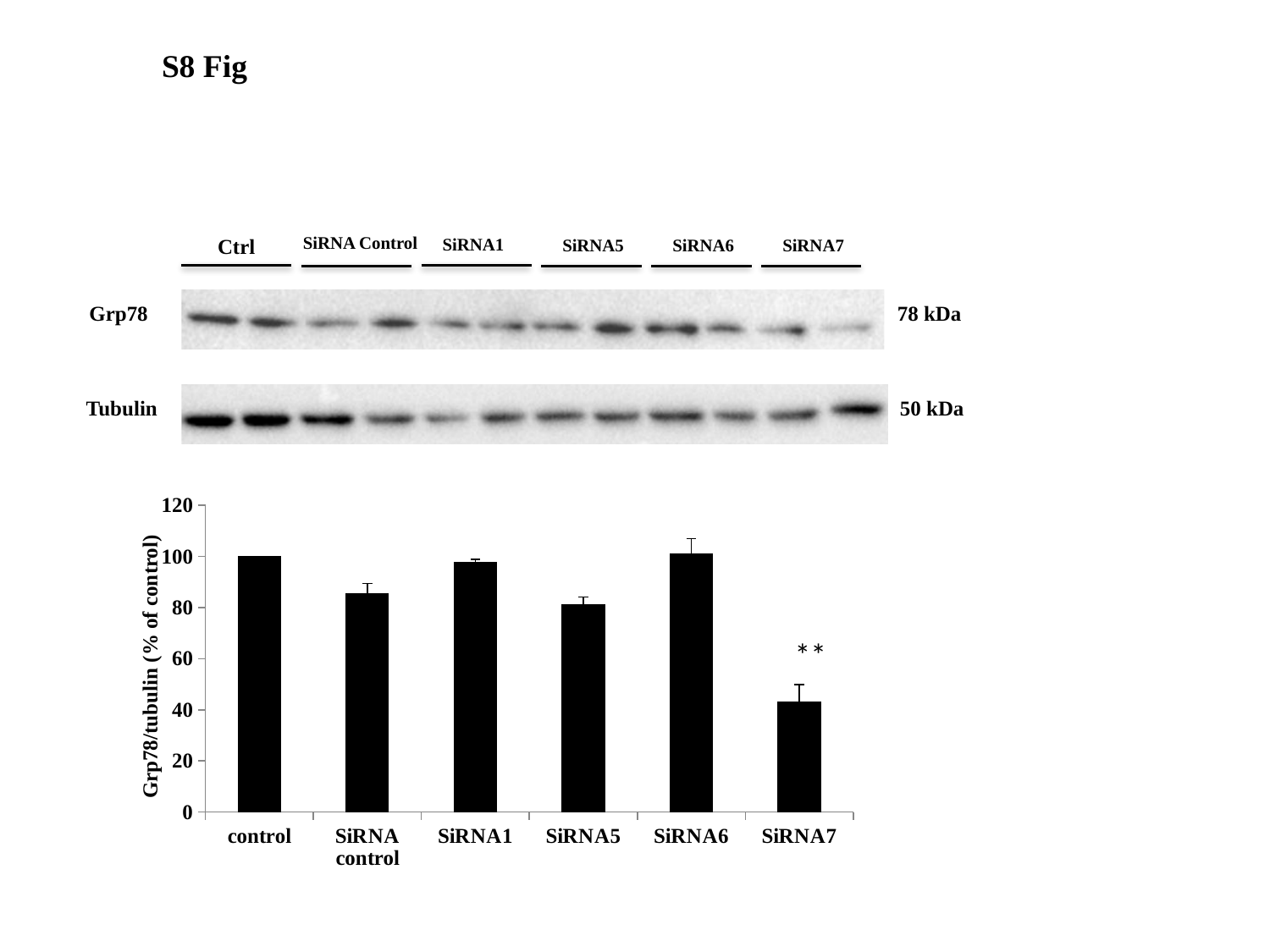

S8 Fig
SiRNA Control
Ctrl
SiRNA1
SiRNA5
SiRNA6
SiRNA7
Grp78
78 kDa
Tubulin
50 kDa
### Chart
| Category | |
|---|---|
| control | 100.0 |
| SiRNA control | 85.50392483776595 |
| SiRNA1 | 97.69694521033773 |
| SiRNA5 | 81.19542145408363 |
| SiRNA6 | 101.0997126841614 |
| SiRNA7 | 43.29045862277767 |Grp78/tubulin (% of control)
**
