# Supplementary material for: Protection of Human Pancreatic Islets from Lipotoxicity by Modulation of the Translocon
Source: PLoS One. 2016 Feb 10;11(2):e0148686. doi: 10.1371/journal.pone.0148686 (PMC4749224; doi:10.1371/journal.pone.0148686)
Supplement: S1 Table — (DOCX) [file pone.0148686.s009.docx]

**S1 Table - Primer sequences used for qRT-PCR**

| **Genes** | **sequence (for/rev)** |
| --- | --- |
| **hATF4** | TGGATGCTCTGTTTCGAATG  AGAATGTAAAGGGGGCAACC |
| **hCHOP** | CTGGAAGCCTGGTATGAGGA  CTCTGACTGGAGTCTGGAG |
| **hGRP78** | CCACCTCCAATATCAACTTG  ACGATCAGGGCAACCGCATCA |
| **hSEC61a** | CTTGATTGCCACCATCTTCG  ACACCAGAGCAGACTGCAAG |
| **hTBP** | TGGTGTGCACAGGAGCCAAG  TTCACATCACAGCTCCCCAC |
| **hXBP1-T** | GTGCAGGCCCAGTTGTCACC  TCTGGGTAGACCTCTGGGAG |
| **hXBP1-S** | CAGACTATGTGCACCTCTGC  TCTGGGTAGACCTCTGGGAG |
